# Autometa: automated extraction of microbial genomes from individual shotgun metagenomes

**DOI:** 10.1093/nar/gkz148

**Published:** 2019-03-06

**Authors:** Ian J Miller, Evan R Rees, Jennifer Ross, Izaak Miller, Jared Baxa, Juan Lopera, Robert L Kerby, Federico E Rey, Jason C Kwan

**Affiliations:** 1 Division of Pharmaceutical Sciences, School of Pharmacy, University of Wisconsin–Madison, 777 Highland Avenue, Madison, WI 53705, USA; 2 Department of Bacteriology, University of Wisconsin–Madison, 1550 Linden Drive, Madison, WI 53706, USA

## Abstract

Shotgun metagenomics is a powerful, high-resolution technique enabling the study of microbial communities *in situ*. However, species-level resolution is only achieved after a process of ‘binning’ where contigs predicted to originate from the same genome are clustered. Such culture-independent sequencing frequently unearths novel microbes, and so various methods have been devised for reference-free binning. As novel microbiomes of increasing complexity are explored, sometimes associated with non-model hosts, robust automated binning methods are required. Existing methods struggle with eukaryotic contamination and cannot handle highly complex single metagenomes. We therefore developed an automated binning pipeline, termed ‘Autometa’, to address these issues. This command-line application integrates sequence homology, nucleotide composition, coverage and the presence of single-copy marker genes to separate microbial genomes from non-model host genomes and other eukaryotic contaminants, before deconvoluting individual genomes from single metagenomes. The method is able to effectively separate over 1000 genomes from a metagenome, allowing the study of previously intractably complex environments at the level of single species. Autometa is freely available at https://bitbucket.org/jason_c_kwan/autometa and as a docker image at https://hub.docker.com/r/jasonkwan/autometa under the GNU Affero General Public License 3 (AGPL 3).

## INTRODUCTION

Microbes are known to associate with almost all organisms on Earth, including humans, where they are thought to have tremendous impact in health, disease and agriculture (1–3). However, it has long been known that only a minute fraction of environmental microbes are readily cultured in the laboratory (4). Thus, the vast majority of the microbial tree of life is as yet only accessible through culture-independent sequencing (‘metagenomics’). Early metagenomic studies focused on phylogenetic profiles of communities by examining the relative abundance of individual bacterial species within different environments (quantified through 16S rRNA gene sequencing), but offered limited information about the functional contribution and organism-level interactions that shape these environments (5). Whole genome ‘shotgun’ sequencing is able to overcome some of the challenges faced by high-throughput 16S rRNA amplicon sequencing, such as the issue with non-canonical ribosomal RNA genes that are undetectable through standard primers (6) and the inherent low-resolution nature of single gene studies. However, the task of sorting metagenomic contigs into clusters representing individual genomes (‘binning’) is a challenging computational problem and an active area of research (7,8). Binning is a necessary step toward understanding the metabolic and functional contributions of individual microorganisms to metabolic capabilities of the community as a whole. In other words, genome-level resolution of metagenomes allows researchers to move beyond the interpretation of metabolic function in aggregate to understanding the role of individual organisms within a complex system *in situ*.

Given that most environments are predominantly composed of uncharacterized microorganisms, different approaches have been taken to achieve reference-free binning. For instance, nucleotide composition has been used to group contigs with emergent self-organizing maps (ESOM) (9) or Barnes-Hut stochastic Neighbor Embedding (BH-tSNE) (10,11). These approaches reduce variation in *k*-mer frequencies to two dimensions, enabling the visualization of highly dimensional data and allowing human-driven clustering. Other efforts have focused on leveraging information from multiple samples, with the assumption that contigs in shared genomes will show a distinct co-variance in coverage. Both manual (12,13) as well as automatic pipelines (14,15) have used this approach. However, there are a number of disadvantages to this methodology. Many multi-sample protocols require assembly of reads from all samples (referred to as ‘co-assembly’), increasing computational requirements and potentially degrading assembly quality when shared genomes are not clonal. This issue is known as ‘microdiversity’—a problem acknowledged by Albertsen *et al.* (13) and recently demonstrated elsewhere (16). By pooling samples for co-assembly, users can also exacerbate the effect of population summing, whereby a genome assembly represents broadly aggregated consensus sequences instead of the genome of a single strain, organism or population taken from one sample (8,16,17). Such aggregation can mask the presence of pan-genome sequences (18) found only in individual strains or samples, which has important clinical and biotechnological implications when considering mobile elements that confer antibiotic resistance (19,20) or biosynthetic gene clusters acquired through horizontal transmission. There are further situations where the underlying variability or overlap of the system is unknown, and there is a desire to extract information from a small number of pilot datasets. Additionally, multi-sample comparisons, which by nature incur higher sequencing costs, do not necessarily aid in binning of genomes unique to one sample (21).

To date, our efforts to sequence the genomes of marine invertebrate symbionts that make bioactive small molecules have relied upon semi-manual binning techniques (21,22). However, marine sponge microbiomes, which offer a wealth of biotechnological potential (23), can contain hundreds of microbial species, occupying up to 40% of the sponge’s tissue volume (24,25). Other systems are also challenging. For example, we found that eggs of the beetle *Lagria villosa* are associated with a mixture of several closely related strains of *Burkholderia gladioli*, but only some of these are culturable and produce antifungal compounds that protect the eggs from infection (26). As these systems were beyond the limit of reasonable manual processing, and due to the poor performance of existing automatic binning pipelines for such host-associated metagenomes, we were motivated to develop an automated and scalable binning algorithm, which we call ‘Autometa’. This method carries out clustering on a simplified subset of contigs (those taxonomically classified as either Bacteria or Archaea), in order to maximize scaling according to metagenomic complexity from individual metagenome assemblies. The initial clusters serve as the training set for subsequent classification by a supervised machine learning algorithm. We evaluated Autometa using a number of simulated and synthetic metagenomes, where performance could be assessed with reference to the known component genomes, as well as a real host-associated metagenome we previously examined by semi-manual binning (21,22). We found that Autometa performed comparably or outperformed MaxBin (14), MetaBAT (27), MyCC (28) and BusyBee Web (29), especially in cases with higher metagenome complexity and in a host-associated dataset. We further found that contig-level taxonomic classification using lowest common ancestor (LCA) analysis was able to improve Autometa’s performance as well as the binning performance of other pipelines.

## MATERIALS AND METHODS

### Overview

**Figure 1.**
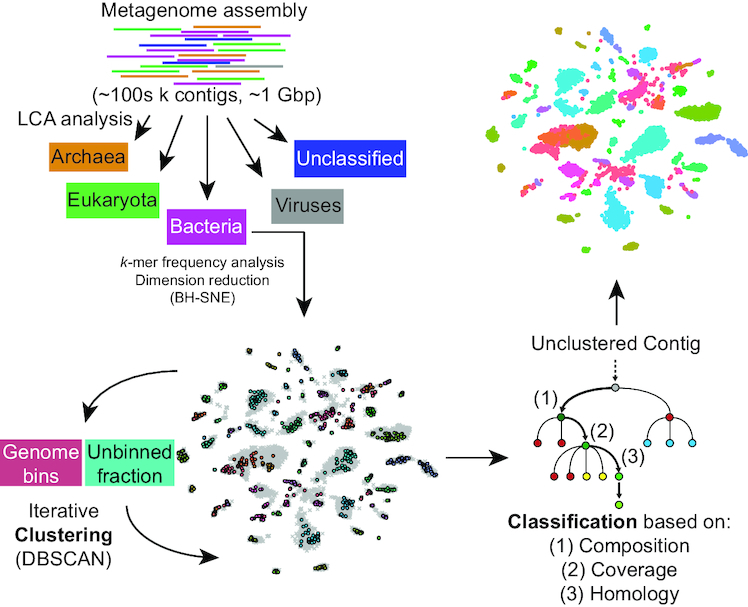
Autometa binning workflow. Autometa separates contigs from a *de novo* metagenome assembly into kingdom-level bins based on sequence homology, iteratively clusters kingdom-specific (Bacterial or Archaeal) contigs, and then (optionally) classifies any remaining unclustered contigs to bins using a decision tree classifier.

Autometa bins microbial genomes *de novo* from single shotgun metagenomes using sequence homology, coverage and nucleotide composition to distinguish between contigs. The task is guided by the presence of marker genes, previously identified in Bacteria and Archaea (30) and known to occur as single copies in microbial genomes. The presence of marker genes can be used to estimate the genome completeness of bins, as well as the level of contamination, as each marker should only be detected once per bin. Single-copy markers have previously been used in MaxBin (14) and MyCC (28), but here we take a different approach. In MaxBin, single-copy markers are used to initialize the number of clusters and their average tetranucleotide frequencies and coverage for an expectation maximization (EM) algorithm (14). A median of ≥2 markers in a bin is used as a crude measure of whether EM has converged. MyCC utilizes single-copy markers after one round of clustering by affinity propagation, to determine which clusters should be merged or split (28). Both of these pipelines suffer from an assumption that true genomes will have the expected number of markers, and in the case of MyCC, this information is not used to guide the clustering step. By contrast, Autometa uses single-copy markers to guide clustering, and does not assume that recoverable genomes will necessarily be ‘complete’. The microbes found in environmental metagenomes can be highly divergent from all previously sequenced organisms, and those that associate with eukaryotic hosts often undergo a process of genome degradation and reduction, where functions essential to independent life can be lost (31,32). For instance, we recently identified a genome-reduced bacterium that was so divergent from known sequences that only 20% of genes had hits in the NCBI NR database, and only 20% of the expected bacterial single-copy markers could be detected (21). We therefore do not assume bins should be close to 100% complete or use single copy markers to pre-calculate the number of bins, as in MaxBin (14). The overall process employed in Autometa comprises three broad stages (Figure 1):

- Separate contigs into kingdom bins based on sequence homology.
- Iteratively cluster kingdom-specific contigs.
- Classify unclustered contigs to bins via supervised machine learning.

### Separation of contigs into kingdom bins

A broad separation of contigs into kingdom bins allows the removal of host-derived or other eukaryotic contamination (even if the host genome is not represented in reference databases), as well as separation of contigs derived from Bacteria and Archaea, simplifying subsequent deconvolution. Genes are identified in all contigs longer than a specified length cutoff with Prodigal (33) (the default is 10 000 bp, but all datasets tested here were based on a 3000-bp cutoff). Translated coding sequences are then queried against the NCBI NR database using the accelerated BLAST implementation Diamond (34). The LCA of the hits with bitscore within 10% of the top hit is used to assign a taxonomy ID to each predicted protein according to the NCBI taxonomy database (https://www.ncbi.nlm.nih.gov/taxonomy). To reduce the influence of horizontally transferred genes, contig-level taxonomy is assigned by a modified majority vote of the component predicted coding sequences. Classifications are considered in order of decreasing specificity (species, then genus, family, order, class, phylum and kingdom), and accepted when a majority (≥50%) classification is reached, provided that the majority of proteins classified with lower specificity are ancestors of this classification. If an answer cannot be reached by this process, the lowest common ancestor of all proteins within a contig is used as the contig classification. Because eukaryotic genomes have low coding density, this system might conceivably lead to incorrect assignment of eukaryotic contigs as bacterial/archaeal in the case of interkingdom horizontal gene transfer (HGT). While a filter for coding density might distinguish most bacterial contigs from eukaryotic ones, employing the wrong cutoff would exclude low-density symbiont genomes at early points in genome reduction (31). Most identified bacterial to eukaryotic HGT events are from organelles (35), and therefore we anticipate that in these cases the closest BLAST hits will be other organelle genes, tied to the host taxonomy. Additionally, the use of prokaryotic gene-finding algorithm Prodigal is expected to yield multiple ORFs for each eukaryotic gene, corresponding to each exon, thus potentially weighting eukaryotic classifications over prokaryotic ones. Very divergent prokaryotic genomes can contain contigs with varying classification even at the phylum level (21), and therefore taxonomic classification is used cautiously in subsequent operations (see below). At this stage, contigs are separated into bins classified according to different kingdoms, and contigs classified as Bacteria and/or Archaea are progressed to the next step.

### Clustering kingdom-specific contigs

It has been shown that *k*-mer frequency patterns differ between bacterial species/strains (9,36), and that visualization of *k*-mer frequency data after dimension reduction with Barnes-Hut Stochastic Neighbor Embedding (BH-tSNE) (37) effectively aids manual deconvolution of metagenomic contigs (10,11). However, the feasibility and throughput of visual (manual) binning using BH-tSNE quickly degrades with increasing metagenome complexity. Autometa counts 5-mer frequencies in contigs, normalizes and reduces the raw dimensions to 50 with principal component analysis (PCA) as previously described (10), before dimension reduction with BH-tSNE.

In BH-tSNE, the parameter of ‘perplexity’ can be conceptualized as the effective number of neighbors considered when the algorithm embeds local structure. In previous work (10), a perplexity value of 30 has been used, so we sought to determine if this was a reasonable value to use in all cases, or whether the parameter should be optimized for different datasets. It has been suggested (38) that a factor referred to as pseudo Bayesian Information Criteria (pBIC, or *S*) might be used to determine the optimum perplexity value (judged by human machine learning experts), where the optimum perplexity gives the minimum value of *S*. In simulated and synthetic metagenomes (see below), we found that minimum *S* values scale with the number of contigs (Supplementary Figures S1– S6).

To determine whether *S* would be a valid parameter for optimizing perplexity, we devised an objective measure of separation based on alignments of metagenomic contigs to the input genomes in simulated/synthetic datasets. For a particular perplexity, we construct groups of points in BH-tSNE space based on their assigned genomes (discounting contigs that are misassembled or unalignable). Within the groups, we discard outliers whose distance away from the group’s centroid is greater than the third quartile of distances plus the interquartile range multiplied by 1.5. From the remaining points, a convex hull is constructed, and we determine both the total area of the hull, *t*, and the area that is not overlapped by any other genome convex hull, *u*. The ‘non-overlapping fraction’, $v$, of the coordinate set for a given perplexity is given by equation (1), where the optimum perplexity should yield the maximum value of $v$, representing the greatest separation between genomes in BH-tSNE space. We plotted $v$ against perplexity for all simulated datasets where a ground truth was known (see below, Supplementary Figures S7– S12). Importantly, peak values of $v$ did not occur at perplexities close to those giving minimum values for *S*, meaning that *S* is a poor predictor for $v$. A degree of variability in adjacent values of perplexity was observed, due to the stochastic nature of BH-tSNE, but the peak value of $v$ generally occurred between perplexities of 20 and 70, regardless of dataset size. As a relationship between dataset size and optimal perplexity was not found, and a method for optimizing perplexity in the absence of ground truth information was not apparent, we use a default value of 30 in the Autometa pipeline.

(1) \begin{equation*} v = \frac{\sum \limits _1^n u}{\sum \limits _1^n t} \times 100 \end{equation*}

Clustering is achieved with the DBSCAN algorithm (39), which clusters based on local density and is able to exclude outliers. In other words, it does not force all contigs into a bin, minimizing the potential for overfitting. DBSCAN has been previously implemented to cluster the output of dimension reduction of pentanucleotide frequencies via BH-tSNE (40). Here, as input to the DBSCAN algorithm, we use the two dimensions produced by BH-tSNE as well as contig coverage. The eps parameter for DBSCAN controls the size of the local neighborhood around each point that is explored during clustering, and we cycle through ascending values of eps from 0.3, increasing by 0.1 until only one group is obtained. For each of these iterations, Autometa assesses clusters by examining both their completeness (number of expected single copy markers) and purity (number of single copy markers that are unique in the cluster). The eps value selected is the one that gives the highest median completeness of bins that are above 20% complete and 90% pure, and the resulting bins that pass these criteria are kept. This method has the advantage that it assesses clustering in a biologically relevant manner, in contrast to internal clustering validation functions (41), and it balances recall (completeness) and precision (purity) of the resulting bins. The clusters that do not meet these criteria and contigs in the ‘unclustered’ bin are then subjected to another round of DBSCAN, again maximizing for median completeness of clusters over 20% complete and 90% pure. This process is iterated until no more clusters meeting the completeness and purity criteria can be obtained. In our investigations of the effects of perplexity on $v$ (Supplementary Figures S7– S12), we found that peak values of $v$ decreased with increasing dataset size, illustrating that BH-tSNE is not able to avoid spatial overlap in complex datasets. In these cases, it is expected that further fractionation of the data based on orthogonal properties will improve clustering quality. Therefore, we allow the unclustered fraction in each iteration to be optionally further divided into taxonomic groups in ascending order of specificity (phylum, then class, order, family, genus and species). After each split, the iterative DBSCAN algorithm described above is repeated, and if unclustered sequences result, they are pooled for clustering at the next specific taxonomic level. This process allows the deconvolution of taxonomically distinct genomes that exhibit similar *k*-mer frequency and coverage, and starts at the non-specific end of the taxonomic spectrum (i.e. phylum before class) to first yield well-separated clusters and to maximize the chance of clustering divergent genomes that exhibit uncertain taxonomic classification (see above).

### Classifying the remaining contigs by supervised machine learning

Following initial clustering of contigs into bins, all remaining (i.e. unclustered) contigs are further recruited to these cores using a supervised decision tree classifier approach. The classifier is trained with features of clustered single copy gene marker-containing contigs, using 5-mer frequencies reduced to 50 dimensions via PCA, as well as sequence coverage, and (optionally) taxonomic information encoded as a binary indicator matrix. The confidence of each of the classifier’s predictions is measured using jackknife cross validation whereby the classifier is iteratively re-trained with a random subset (50%) of the training data (42). By default, a prediction will only be accepted if this metric reports 100% confidence (e.g. 10/10 consistent classifications when trained with 10 random subsamples of the training data) and the prediction does not add any marker contamination to the predicted bin. After each full round of predictions, any marker-containing contig that is confidently classified to pre-existing clusters is added to the training data for subsequent rounds of classification, until no further marker-containing contigs are confidently classified. This approach is similar to the ‘bootstrapping’ of supervised machine learning in BusyBee Web using the result of unsupervised clustering, except that it includes features beyond nucleotide composition, such as sequence coverage and taxonomic information in the prediction process and uses jackknife cross validation (Supplementary Figure S13) to assess the confidence of each prediction.

### Implementation

Autometa is implemented in Python, and the source code is available at https://bitbucket.org/jason_c_kwan/autometa. The pipeline is run through the command line, and has been tested on various Linux distributions. Full documentation on installation, dependencies, etc. is provided with the code repository, and we have also built a Docker image (available at https://hub.docker.com/r/jasonkwan/autometa) to facilitate easy installation and reproducible analyses.

### Benchmarking datasets

Simulated metagenomes of increasing complexity (see Table 1) were created by picking random genomes out of the bacterial genome assemblies held in the NCBI database. Illumina reads (2 × 125 bp) were simulated using ART (43) and assembled with metaSPAdes (v3.9.0) (44). A script included with Autometa (‘make_simulated_metagenome.py’) was used to generate these test datasets, automating assembly retrieval and read simulation of randomly selected bacterial genomes. This script used art_illumina parameters: -p -ss HS25 -l 125 -m 275 -s 90, and the default parameters for metaSPAdes (i.e. SPAdes was run with the - -meta flag). Datasets were simulated to represent each component genome with equal coverage in order to stress-test binning performance based on nucleotide composition. Five synthetic metagenomes were also prepared. ‘Mix-51’ was made by mixing together roughly equal amounts of cell pellets from 51 bacteria isolated from the human gut, before extracting DNA. For the remaining four synthetic metagenomes, DNA was separately extracted from the 51 bacterial isolates and quantified. Two DNA solutions of Mix-51 were prepared at equimolar and differential concentrations (‘Mix-51-equal’ and ‘Mix-51-staggered’), respectively (see Table 2 and Supplementary Table S1). An environmental metagenome from a sample of the marine sponge *Hippospongia lachne* (termed ‘FL20-9’) was spiked into one of each of the synthetic metagenomes resulting in ‘FL20-9-Mix-51-equal’ and ‘FL20-9-Mix-51-staggered’ (see Supplementary Table S1). DNA pellets for each of the mixtures or strains were dissolved in TE buffer (10 mM Tris–HCl pH 8.0, 1 mM ethylenediaminetetraacetic acid) then column-purified using the Nucleospin Gel and PCR Clean-up kit (Macherey-Nagel Inc, Bethlehem, PA). DNA extractions were performed as previously described (45). The DNA concentration of Mix-51 was measured using the Qubit BR dsDNA assay (Invitrogen, Eugene, OR). DNA from the separate bacterial isolates and the sponge metagenome were quantified using the Quant-iT PicoGreen dsDNA assay kit (Life Technologies, Eugene, OR). Sequencing of Mix-51 DNA was carried out on an Illumina HiSeq 2500, in a 2 × 125 bp run. Sequencing of the concentration-controlled Mix-51 samples was carried out on an Illumina NovaSeq 6000, in 2 × 150 bp runs. Adapters were trimmed from the resulting reads using Trimmomatic (46), before being assembled with metaSPAdes (44). Further information on the datasets, including details of component genomes, can be found in Table 2 and Supplementary Table S2.

**Table 1.** Datasets used in this study

Dataset	Type	No. genomes	*N*_50_ (bp)^a^	Assembled length (Mbp)^a^
78.125 Mbp	Simulated	23	96 188	78.0
156.25 Mbp	Simulated	42	150 368	156.2
312.5 Mbp	Simulated	85	123 776	297.2
625 Mbp	Simulated	157	139 531	607.8
1250 Mbp	Simulated	341	106 210	1,193.2
2500 Mbp	Simulated	650	59 220	2,217.4
5000 Mbp	Simulated	1,308	7222	3,179.1
10000 Mbp	Simulated	2,617	4611	427.5
Mix-51	Synthetic	51	133 668	184.6
Mix-51-equal	Synthetic	51	114 621	188.5
Mix-51-staggered	Synthetic	51	46 545	160.1
FL20-9-Mix-51-equal	Synthetic Host-Associated	223^b^	8810	1224
FL20-9-Mix-51-staggered	Synthetic Host-Associated	223^b^	7668	1209
AB1_ovicells	Host-Associated	8^c^	11 056	237.6

^a^For contigs ≥3 kbp.

^b^Includes 51 genomes in synthetic mixture and 172 additional genome bins in the sponge metagenome, obtained through Autometa analysis of the non-spiked metagenome.

^c^As previously identified in Miller *et al.* (21).

**Table 2.** Component genomes of Mix-51 datasets

Strain	Accession	Genome size (bp)	Status	Attomoles in Mix-51-staggered^a^
*Alistipes indistinctus* YIT 12060	GCA_000231275.1	2 855 429	Draft	4
*Bacteroides cellulosilyticus* DSM 14838	GCA_000158035.1	6 870 144	Draft	200
*Bacteroides finegoldii* DSM 17565	GCA_000156195.1	4 892 401	Draft	20
*Bacteroides intestinalis* DSM 17393	GCA_000172175.1	2 642 081	Draft	120
*Bacteroides ovatus* ATCC 8483	NZ_CP012938.1	6 465 369	Complete	60
*Bacteroides plebeius* DSM 17135	GCA_000187895.1	4 421 924	Draft	80
*Bacteroides stercoris* ATCC 43183	GCA_000154525.1	4 009 829	Draft	240
*Bacteroides thetaiotaomicron* 3731		7 187 176	Complete	160
*Bacteroides thetaiotaomicron* 7330	GCA_001314975.1	6 487 685	Complete	320
*Bacteroides thetaiotaomicron* VPI-5482	GCA_000011065.1	6 293 399	Complete	40
*Bacteroides uniformis* ATCC 8492	GCA_000154205.1	4 719 097	Draft	280
*Bacteroides vulgatus* ATCC 8482	GCA_000012825.1	5 163 189	Complete	400
*Bacteroidetes dorei* DSM 17855	GCA_000156075.1	5 566 217	Draft	4
*Bifidobacterium adolescentis* L2-32	GCA_000154085.1	2 389 110	Draft	4
*Bifidobacterium angulatum* DSM 20098	NZ_AP012322.1	2 008 208	Complete	60
*Bifidobacterium bifidum* ATCC 29521	NZ_AP012323.1	2 201 251	Complete	80
*Bifidobacterium dentium* ATCC 27678	GCA_000172135.1	2 642 081	Draft	40
*Bifidobacterium pseudocatenulatum* DSM 20438	GCA_000173435.1	2 304 808	Draft	20
*Blautia hansenii* DSM 20583	NZ_CP022413.2	3 058 721	Complete	8
*Blautia luti* DSM 14534		4 068 430	Complete	32
*Citrobacter youngae* ATCC 29220	GCA_000155975.1	5 154 159	Draft	64
*Clostridium asparagiforme* DSM 15981	GCA_000158075.1	6 417 332	Draft	64
*Clostridium bolteae* ATCC BAA-613	NZ_CP022464.2/NZ_CP022465.2	6 557 988	Complete	8
*Clostridium hathewayi* DSM 13479	GCA_000160095.1	7 163 884	Draft	32
*Clostridium hylemonae* DSM 15053	GCA_000156515.1	3 889 859	Draft	16
*Clostridium ramosum* DSM 1402	GCA_000154485.1	3 235 195	Draft	16
*Clostridium* sp. M62/1	GCA_000159055.1	3 842 594	Draft	64
*Clostridium sporogenes* ATCC 15579	GCA_000155085.1	4 102 325	Draft	8
*Clostridium symbiosum* ATCC 14940	GCA_000466485.1	4 823 675	Draft	32
*Collinsella intestinalis* DSM 13280	GCA_000156175.1	1 809 497	Draft	16
*Collinsella stercoris* DSM 13279	GCA_000156215.1	2 475 429	Draft	16
*Coprococcus comes* ATCC 27758	GCA_000155875.1	3 242 215	Draft	8
*Dorea formicigenerans* ATCC 27755	GCA_000169235.1	3 186 031	Draft	32
*Edwardsiella tarda* ATCC 23685	GCA_000163955.1	3 744 568	Draft	8
*Enterobacter cancerogenus* ATCC 35316	GCA_000155995.1	4 638 653	Draft	4
*Escherichia fergusonii* ATCC 35469	GCA_000026225.1	4 643 861	Complete	16
*Eubacterium biforme* DSM 3989	GCA_000156655.1	2 517 763	Draft	4
*Eubacterium eligens* ATCC 27750	GCA_000146185.1	2 831 389	Complete	16
*Holdemania filiformis* DSM 12042	GCA_000157995.1	3 932 923	Draft	4
*Lactobacillus reuteri* DSM 20016	GCA_000016825.1	1 999 618	Complete	4
*Lactobacillus ruminis* DSM 20403	GCA_001436475.1	2 008 484	Draft	32
*Marvinbryantia formatexigens* DSM 14469	GCA_000173815.1	4 548 960	Draft	64
*Megamonas funiformis* YIT 11815	GCA_000245775.1	2 562 512	Draft	4
*Parabacteroides johnsonii* DSM 18315	GCA_000156495.1	4 787 097	Draft	80
*Parabacteroides merdae* ATCC 43184	GCA_000154105.1	4 434 377	Draft	40
*Proteus penneri* ATCC 35198	GCA_000155835.1	3 749 229	Draft	8
*Roseburia intestinalis* L1-82	GCA_000156535.1	4 411 375	Draft	64
*Ruminococcus gnavus* ATCC 29149	GCA_000169475.1	3 501 911	Draft	8
*Streptococcus infantarius* ATCC BAA-102	GCA_000154985.1	1 925 187	Draft	64
*Subdoligranulum variabile* DSM 15176	GCA_000157955.1	3 245 471	Draft	16
*Tyzzerella nexilis* DSM 1787	GCA_000156035.2	3 995 628	Draft	4

^a^Based on the assumption that draft-quality genomes represent the true genome size. As discussed in the main text, under ‘Performance in synthetic metagenomes with highly similar strains’, coverages in the resulting assembled metagenomes suggested that calculated molar quantities of draft genomes were inaccurate due to uncertainty in the genome sizes.

We also included sample AB1_ovicells in benchmarks, which we previously examined semi-manually (21,22). This is a metagenome associated with a marine bryozoan, containing the uncultured bryostatin-producing symbiont, ‘*Candidatus* Endobugula sertula’ along with several divergent bacteria and several genomes that are very similar in GC content and/or coverage. The same assembly used previously (21,22) was assessed as a point of comparison to manual binning efforts. All datasets were tested using Autometa commit version 9592e35 and run on a linux server (Dell Poweredge T430 with two Intel Xeon E5-2650 v3 2.3 GHz CPUs, 128 GB of RAM and 1.7 TB of disk space).

## RESULTS

### Benchmarking approach

#### Choice of comparison pipelines

To enable an apposite comparison of Autometa’s performance with existing pipelines, we excluded pipelines with different aims, such as those designed to pre-cluster raw sequence reads or those that required multiple metagenomic datasets. We also excluded pipelines that required manual interpretation of visualizations, on the grounds that these did not include an automated clustering step. This rationale led us to focus on four pipelines for comparison: MaxBin (14), MetaBAT (27), MyCC (28) and BusyBee Web (29).

#### Evaluation metrics

AB1_ovicells is a real dataset associated with the adult bryozoan *Bugula neritina*. In previous investigations (22), we sought to assemble the vertically transmitted symbiont ‘*Candidatus* Endobugula sertula’ by identifying non-host contigs with >1× coverage in both AB1_ovicells and the metagenome of free-swimming larvae. Therefore, because the complete ground truth in AB1_ovicells is unknown, we assess the results with reference to conservation in both the adult and larval samples in the case of ‘*Ca*. E. sertula’ and by using estimates of completeness and purity by CheckM (47) for other bacterial species present. In the case of the simulated metagenomes and Mix-51 derivatives, contigs were assigned to reference genomes with MetaQUAST (48). Precision and recall were then calculated as described previously (28) according to equations (2) and (3), where we consider the binning of *N* genomes into *M* clusters and *S*_*ij*_ is the combined length of contigs in cluster *i* which belong to reference genome *j*. Precision is a property of clusters, described as the length fraction of a cluster taken up by contigs belonging to the genome accounting for the largest length fraction of the cluster (max*_j_*). Recall is a property of reference genomes, described as the length fraction of the genome assigned to the cluster with the largest fraction of that genome (max_*i*_). For the purposes of these calculations, contigs labeled as ‘misassembled’ by MetaQUAST were excluded. The F1 score is the harmonic mean of precision and recall (equation 4). Note here that in order to distinguish the effects of assembly from binning, a perfect F1 score is achieved when all assembled contigs from a given genome are assigned to a single bin.

(2) \begin{equation*} \mathrm{Precision} = \frac{\sum _{i=1}^{M} {\rm max}_{j} S_{ij}}{\sum _{i=1}^{M} \sum _{j=1}^{N} S_{ij}} \times 100 \end{equation*}

(3) \begin{equation*} \mathrm{Recall} = \frac{\sum _{j=1}^{N} {\rm max}_{i} S_{ij}}{\sum _{i=1}^{M} \sum _{j=1}^{N} S_{ij} + \sum \mathrm{unbinned}} \times 100 \end{equation*}

(4) \begin{equation*} \mathrm{F1} = 2 \times \frac{\mathrm{Precision} \times \mathrm{Recall}}{\mathrm{Precision} + \mathrm{Recall}} \end{equation*}

### Performance in a host-associated metagenome

**Figure 2.**
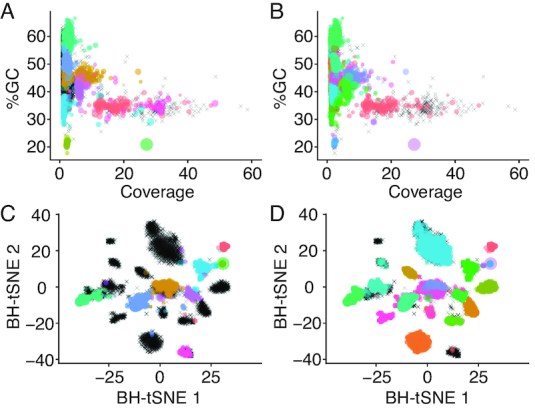
Visualization of genome bins from a host-associated metagenome derived from semi-manual binning (left column, (**A**) and (**C**)) versus automated binning (right column, (**B**) and (**D**)). Points represent contigs and are colored based on their assigned bin with size scaled by length; unclustered contigs are represented by black crosses. In the top row ((**A**) and (**B**)), contigs are plotted on axes of GC% and coverage, while in the bottom row ((**C**) and (**D**)), contigs are plotted on the two dimensions derived from dimension-reduction of 5-mer frequencies by Barnes-Hut Stochastic Neighbor Embedding.

Compared to our previous semi-manual binning efforts for the AB1_ovicells sample, all four tested programs produced a greater number of bins. Autometa produced 22 genome bins (Table 3) compared to the eight we identified by our earlier, semi-manual approach (Figure 2 and Table 1). However, when comparing the performance of these programs to the composition of manually classified sequences, the bin-level performance was more variable. For instance, each program performed differently when compared to our semi-manual classification of ‘*Ca*. Endobugula sertula’ contigs. We defined the original AB1_ovicells ‘*Ca*. E. sertula’ assembly to include 3.32 Mbp in 117 contigs that also had coverage in the larval ‘MHD_larvae’ metagenome (22). The cluster statistics of the ‘*Ca*. E. sertula’ bin as identified by the four different programs are detailed in Supplementary Table S3. Autometa produced the genome bin most consistent with semi-manual binning (recovering 92/117 contigs (93.3% of length) derived from semi-manual binning). MaxBin had the second highest recovery of the original ‘*Ca*. E. sertula’ assembly, at a 91.9% recovery rate. Autometa and MaxBin were tied for the highest apparent completeness for this cluster, at 96.2% (as assessed by CheckM (47)). The ‘*Ca*. E. sertula’ cluster identified by MyCC had a slightly higher purity (98.2% compared to 96.6%, according to CheckM results), but with the lowest completeness (71.6%). Interestingly, both MyCC and Autometa identified a shared set of 44 contigs, within the ‘*Ca*. E. sertula’ bin, that we had previously left unclassified through our semi-manual efforts. The nucleotide composition of these contigs was consistent with contigs we identified as belonging to ‘*Ca*. E. sertula’ (Supplementary Figure S14), but had lower sequence coverage on average (Supplementary Figure S15). However, 12 of these 44 contigs (27%) identified by Autometa and MyCC were assigned the order level taxonomy of ‘Oceanospirillales,’ which suggests contamination from another gammaproteobacterial genome bin that we previously identified as an *Endozoicomonas* sp. (Supplementary Figure S16).

**Table 3.** Effect of taxonomic partitioning on binning performance of AB1_ovicells

	AB1_ovicells				AB1_ovicells			
	Without taxonomic filtering				With taxonomic filtering			
Algorithm	No. bins	Median completeness^a^	Median purity^a^	Bacterial fraction (%) ^b^	No. bins	Median completeness^a^	Median purity^a^	Bacterial fraction (%) ^b^
**Autometa**	20	40.9	99.1	97.7	22	34.0	99.6	100
**MyCC**	22	41.1	96.3	24.7	25	31.0	98.8	100
**MaxBin**	27	23.7	95.6	28.5	13	74.3	88.1	100
**MetaBAT**	40	13.2	100.0	24.6	25	23.6	99.9	100

^a^Estimated based on analysis by CheckM (47).

^b^Fraction of the total binned length classified under kingdom Bacteria by Autometa’s LCA pipeline.

The aggregate binning results for Autometa and MyCC for the AB1_ovicells sample appear comparable in the number of bins recovered, along with median purity and completeness metrics, with an apparent tradeoff between purity (higher with Autometa) and completeness (higher with MyCC). However, it is worth noting that CheckM does not systematically consider contamination from host Eukaryotic sequences in its reported contamination statistics and much of the sequence clustered by MyCC, MaxBin and MetaBAT appears contaminated with Eukaryotic sequence (Supplementary Table S4). In fact, at least two MyCC clusters (Cluster.8 and Cluster.2, Supplementary Table S5) appear heavily contaminated by host bryozoan sequence, though CheckM reports their purity as 87.5% and 77.3% and marker lineage as Archaea (Supplementary Table S5). These clusters represent 136.3 and 31.7 Mbp, in 16 825 and 2811 contigs, respectively (Supplementary Table S5). Analysis with Autometa’s LCA workflow suggests only 3.5 Mbp (2.5%) and 1.8 Mbp (5.8%), respectively, of these MyCC clusters are represented by prokaryotic sequence (Supplementary Table S6).

Thus, to test the effect of taxonomic filtering on the binning performance of other pipelines, we repeated runs with MaxBin, MetaBAT and MyCC on just contigs that Autometa’s LCA workflow identified as bacterial. This taxonomic filtering step resulted in decreased bin fragmentation for MaxBin and MetaBAT. In addition to preventing putative host sequences from populating MyCC bins, taxonomic filtering improved the median cluster statistics for MetaBAT and MaxBin. Without taxonomic filtering, MetaBAT identified 40 genome bins with a median completeness of 13.2, compared to the 25 bins identified with a median completeness of 23.6 with taxonomy-filtered contigs (Table 3). Taxonomic filtering also resulted in a consolidation of bins produced by MaxBin, with a concomitant increase in median completeness (74.3% with and 23.7% without taxonomic filtering).

### Performance in synthetic metagenomes with highly similar strains

We first sought to quantify the differing composition of the Mix-51 assemblies, and the resulting effects on assembly quality. Quality-filtered reads from the respective dataset were aligned to the 51 reference genomes. We quantified coverage of each genome by discarding reads that aligned to more than one genome and/or more than one location in a single genome. The length-weighted average coverage of the portion of resulting alignments with coverage >0 is presented in Supplementary Table S7. The average genome coverage is similar for all Mix-51 datasets, but unexpectedly the standard deviation for coverage is the smallest for Mix-51, where roughly equal amounts of cells were mixed prior to DNA extraction. The coverage standard deviation is smaller in Mix-51-equal versus Mix-51-staggered (104 and 201 respectively), but there is higher than expected variation in the former. We suspect that this variation could have resulted from the sizes of draft-quality NCBI genomes being inaccurate, which affected the molar quantities of DNA added to the mixtures. Typically, draft-quality genomes do not accurately reflect the true length of chromosomes, including repeats, and it is not possible to infer the relative copy numbers of plasmids versus chromosomes from sequence alone. GC biases in the amplification step of Illumina sequencing could also contribute to this coverage variation. Importantly, for our purposes, many of the 51 component genomes vary in coverage across the Mix-51 datasets, providing a realistic test for our algorithm. We also combined both Mix-51-equal and Mix-51-staggered with metagenomic DNA from a marine sponge, ‘FL20-9’. In analysis of this marine sponge metagenome, which will be reported elsewhere, Autometa was able to yield 172 bacterial genome bins. We spiked in Mix-51 mixtures and sequenced to a depth that achieved similar coverages for the 51 input genomes in both the synthetic and sponge-spiked metagenomes (Supplementary Table S7). The assembly quality of genomes in Mix-51 was not appreciably affected by mixture with FL20-9, in terms of the percentage of the reference genome assembled *de novo*, with the exception of genomes that were low coverage in Mix-51-staggered compared to Mix-51-equal and Mix-51. Granular examination of assembly quality (i.e. number of contigs, assembly length, *N*_50_, longest contig length) showed that mixture of Mix-51-equal with FL20-9 did not appreciably affect assembly (Supplementary Table S8). In fact, a number of genomes were better assembled in FL20-9-Mix-51-staggered versus Mix-51-staggered, perhaps due to slightly higher coverage. For instance, *Alistipes indistinctus* contigs were found at 5.6× coverage in Mix-51-staggered and 9.9× coverage in FL20-9-Mix-51-staggered, and there is a marked increase in assembly quality in the latter (59 contigs, *N*_50_ 99 kbp versus 756 contigs and *N*_50_ 5 kbp in Mix-51-staggered). This suggests that coverage affected assembly quality by metaSPAdes more than the complexity of the mixture of synthetic and sponge metagenomes. Some genomes also appeared to be poorly assembled by virtue of having close relatives in the mixture (i.e. only small portions of their genomes with uniquely aligned reads), such as *Bacteroidetes thetaiotaomicron* strains VPI-5482 and 3731. Overall, the most important factors determining assembly quality appeared to be coverage and presence of related strains.

**Figure 3.**
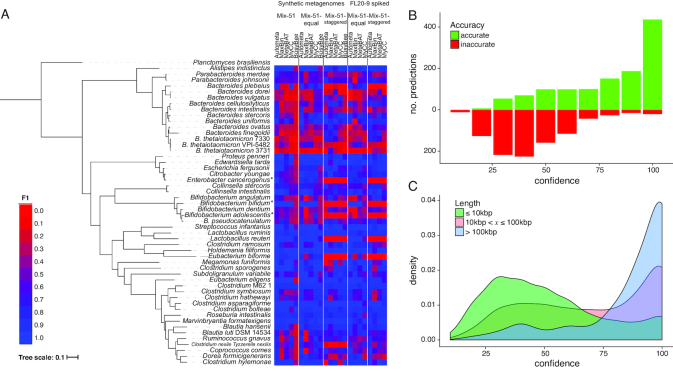
Performance testing and proof of concept of machine learning classification using synthetic metagenomes with high coverage and strain overlap. (**A**) F1 values of individual genomes in Mix-51 datasets as compared to phylogeny based on concatenated protein marker alignments using AMPHORA2 (49). *MetaQUAST failed to assign any contigs >3 kbp in length to *Enterobacter cancerogenus* and *Bifidobacterium adolescentis* for Mix-51-staggered, as well as to *Bifidobacterium bifidum* in both Mix-51-staggered and FL20-9-Mix-51-staggered. These genomes have been assigned F1 scores of zero for the respective datasets. (**B**) Number of accurate and inaccurate predictions compared to the confidence (based on jackknife cross validation, Supplementary Figure S13) of the decision tree classifier when the known reference genomes of single copy marker-containing contigs are provided as a ground truth. In other words, when the model is provided accurate assignments of the marker-containing contigs, its confidence is well correlated to its accuracy (Pearson correlation coefficient for the percent of accurate predictions is 0.9887931, *P* = 6.809 × 10^−8^). (**C**) Density plot showing confidence of the classifier’s predictions compared to the length of the contig being classified.

F1 scores of all 51 input genomes were quantified with respect to the assembled fraction in the respective five Mix-51 assemblies, for each of the five tested algorithms. Contigs in each assembly were assigned to one of the 51 bacterial reference genomes with MetaQUAST (48), and the identified contigs were used as the basis for calculating F1. Autometa consistently yielded the most bins with the highest ranking F1 scores for specific input genomes (Supplementary Table S9). We also quantified the median F1 of all obtained bins, and additionally F1 recovery (the sum of all F1 scores for each genome bin divided by the theoretical maximum sum; Supplementary Table S10). Autometa consistently scored the highest median F1 in all Mix-51 datasets (Supplementary Table S10), and the highest F1 recovery in all datasets except Mix-51-staggered, where MetaBAT had a slightly higher score. All tested algorithms were challenged by the high strain overlap of the synthetic Mix-51 community (Figure 3 and Supplementary Table S9). Scores for spiked-in metagenomes were broadly similar to the corresponding synthetic mixture, except that Autometa was able to exclude eukaryotic contigs from bins. Bins produced by MyCC, Maxbin and MetaBAT were contaminated with eukaryotic sequences (Supplementary Figure S17). All algorithms struggled when there were multiple related strains present, to a varying extent, as evidenced by the concentration of low F1 scores with tight clades (Figure 3). The performance of the algorithms in closely related genomes appeared to be modulated by the relative abundance of the components, as evidenced by differing performance in different Mix-51 datasets. However, patterns did not adhere strictly to the principle that differing coverage of related genomes equates to higher F1. For example, Autometa yielded moderate results for the *Bifidobacterium* strains in Mix-51-equal, despite the fact that coverage of each strain was fairly similar (25–38×, Supplementary Table S7). Performance in these strains was far worse in Mix-51-staggered, where coverages were much lower (1–18×) and assembly quality was lower (Supplementary Table S8). This pattern indicates that although we have endeavored to minimize the effects of assembly on binning results, a decrease in assembly quality ultimately translates to considerably degraded bins.

In addition to stress-testing these automated binning programs with high-strain overlap, the Mix-51 sample was used to validate the performance of the machine learning classification step as a proof of concept. When contigs containing single copy marker contigs (30) were used to train the decision tree classifier (with known reference genomes—as annotated by metaQUAST alignment—provided as labels), the classifier was able to predict the genome identity of other contigs with very high accuracy, where predictions were reported to have high confidence values. There was a strong correlation between the classifier’s confidence (as determined by a jackknife cross-validation approach (Supplementary Figure S13) and the percent of accurate predictions (Figure 3B; Pearson Correlation Coefficient, 0.9887931, *P* = 6.809 × 10^−8^). For instance, 95.4% (436/457) of predictions with 100% confidence were accurate, recruiting 17 Mbp of sequence. On the other hand, only 18.3% (131/714) of predictions with <50% confidence were accurate. It also appears that the confidence of the classifier is positively associated with sequence length (Figure 3C), likely because the signal and resolving power of *k*-mer frequency is known to improve with sequence length (8). The median confidence of predictions for contigs >100, 10–100 and <10 kbp were 90%, 70% and 50%, respectively (Figure 3C).

### Performance in uniform coverage simulated metagenomes

Due to the innate complexity of some marine invertebrate associated microbial communities, such as marine sponges (23–25), we tested the scalability of composition and homology-based techniques for single sample binning analysis. To this end, we tested our algorithm along with MyCC, MaxBin, MetaBAT and BusyBee Web using a set of increasingly complex simulated sequence sets with uniform coverage (Table 1).

**Figure 4.**
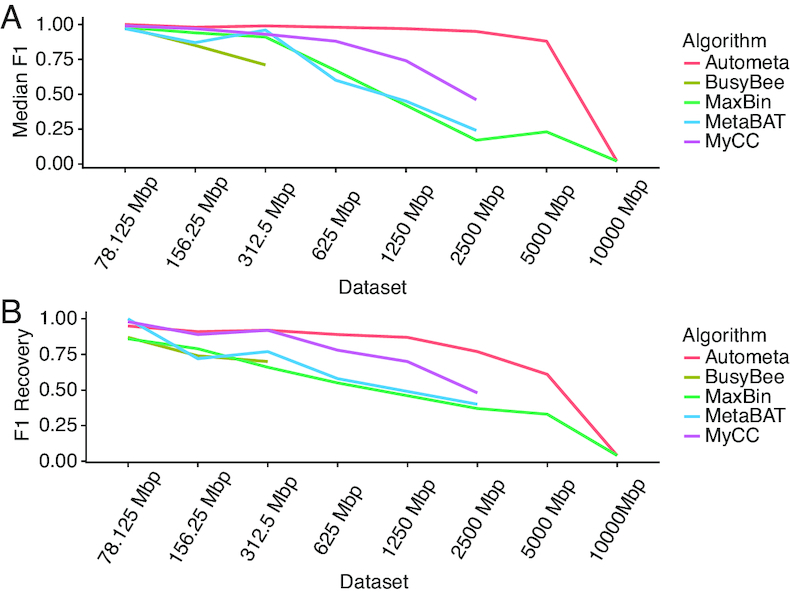
Performance in increasingly complex simulated metagenomes with uniform sequence coverage profiles based on median F1 (**A**) and F1 recovery (**B**).

For each of the simulated datasets, Autometa was able to recover more genome bins (Supplementary Table S12), including in the largest tested dataset (10 000 Mbp), which represented the simulated sequencing of a metagenome containing 2617 bacterial genomes. At the same time, the median F1 score of bins yielded by Autometa is consistently close to 1.0 (Supplementary Table S13), up to and including the 5000 Mbp dataset (Figure 4A). It should be noted that MaxBin was the only pipeline other than Autometa able to complete successfully for the two largest datasets (5000 and 10 000 Mbp). In the smaller datasets (78.125, 156.25 and 312.5 Mbp), the performance of other pipelines is comparable to Autometa, but their performance rapidly declines in more complex datasets. We also calculated F1 recovery for all pipelines (Figure 4B and Table 1). Based on this metric, Autometa and MyCC performed comparably for the three smallest datasets. However, for the larger datasets (625 to 10 000 Mbp) Autometa consistently outperformed MyCC. MaxBin, MetaBAT and BusyBee Web underperformed Autometa and MyCC in all datasets except for the smallest (78.125 Mbp) by this measure, where MetaBAT scored higher (Supplementary Table S14).

It is worth noting that while Autometa’s performance appears to drop dramatically after the 5000 Mbp dataset, this drop is most likely a result of the sharp decline in the assembly quality (Table 1), whereby the *N*_50_ (for contigs ≥3 kbp) drops from 7222 to 4611 bp, and where the total assembled length drops from 3179.1 to 427.5 Mbp (for contigs ≥3 kbp), for the 5000 and 10 000 Mbp datasets, respectively. It is possible that if simulated sequencing parameters were adjusted to simulate greater sequencing depth, the quality of the assembly and thus binning results would continue to scale.

## DISCUSSION

Shotgun sequencing coupled with genome binning enables species-level resolution of metagenomes, even when the genomes of their microbial constituents lack representatives in reference databases. This reference-free approach is moving the field of microbiology from phylogenetic profiling of communities and aggregate interpretation of metagenomic data to a higher resolution perspective of which organisms play particular roles in a given ecosystem. Such information can be invaluable in a diverse array of biotechnological applications, such as identifying the source of bioactive secondary metabolites in complex marine invertebrate communities (17,50,51) or antibiotic resistance mechanisms (20,52–55) in uncultured clinical samples (56,57). However, despite the advances stemming from this paradigm shift in metagenomic analysis, a number of challenges remain.

Many available automated binning programs require the use of multiple samples in order to bin contigs into genome bins based on differential coverage profiles. However, this type of sample collection strategy is often not possible for marine invertebrate communities with dynamic compositions (21), and can be too costly for exploratory sequencing studies (17). Furthermore, these techniques typically rely on the use of co-assemblies, a strategy whereby reads from multiple samples are pooled prior to assembly. This approach leads to increasingly aggregate and chimeric representation of sequences and has been shown to reduce the overall genome assembly quality of constituent genomes (16), which, in turn, reduces the accuracy of the genome binning process. Here, we instead focussed on maximizing the binning performance that can be achieved from single samples, and in subsequent efforts we will aim to capitalize on multiple metagenomes, where available, without resorting to co-assembly and with awareness of intersample strain variability.

In a recent review by Sangwan *et al.*, the authors cited a general lack of binning strategies that integrate phylogenetic analysis with nucleotide composition (8). Part of the challenge in analyzing non-model host-associated microbiomes is the fact that most eukaryotic hosts lack any type of reference genome, and thus, unlike studies of the human microbiome, resulting reads from shotgun sequencing cannot be easily separated from these sequencing datasets using alignment techniques. Much of our approach in developing Autometa aimed to both address this fundamental issue and to further leverage contig-level taxonomic assignments to improve the binning process. Other efforts have focused on removing prokaryotic contamination following *de novo* assembly efforts of eukaryotic genomes (58). Here we have implemented kingdom-level taxonomic partitioning prior to binning, in addition to incorporating taxonomic information in clustering and classification steps, improving binning performance both for Autometa and for other tested binning pipelines in the bryozoan metagenome tested here.

The necessity for genome binning ultimately stems from the underlying shortcomings of modern sequencing technology (8,17), especially in regards to the trade off between read length, accuracy and sequencing depth (59). Thus, short-read sequencing technologies, such as Illumina, are the only platforms currently capable of delivering sufficient sequencing depth and per-read accuracy to effectively assemble low abundance genomes directly from host-associated metagenomes without physical or chemical enrichment of bacterial DNA (60), which can introduce unforseen sampling bias. As the throughput and accuracy of longer read technologies continue to advance, the demands for binning strategies could feasibly decrease. However, for the foreseeable future, genome-resolved metagenomics will rely on contig-binning strategies based on a combination of coverage, composition and homology. No individual binning model will likely be able to outperform all others under every circumstance. Furthermore, there are fundamental limitations of binning sequences based on coverage, composition, and homology features that complicate the proper assignment of mobile elements such as plasmids and genes acquired through horizontal transmission, especially when they are poorly assembled (for instance, due to high repeat content). Thus, it is important that users understand the assumptions of each approach (7,8) and interpret results accordingly. Others have suggested and demonstrated that a combined strategy, particularly using programs with distinct underlying algorithms, is most likely to yield the most robust results (8,61,62). We have shown here, however, that the integration of taxonomic information with nucleotide composition in Autometa allows it to outperform several other pipelines in host-associated and extremely complex metagenomes, yielding hundreds of high-quality genome bins from single datasets. This capability will complement existing multi-sample techniques by allowing the analysis of inter-sample strain variability in high resolution, which is likely to be seen in vertically transmitted symbionts.

## DATA AVAILABILITY

The raw reads of the Mix-51-derived datasets are accessible through the Sequence Read Archive (SRA), under accession numbers SRR5679054, SRR8304764–67, SRR8304769–76 and SRR8304783–86. Previously published raw reads and annotated assemblies associated with sample AB1_ovicells are available through NCBI (BioProject PRJNA322176).

## Supplementary Material

gkz148_Supplemental_Files
