# Supplementary material for: Autometa: automated extraction of microbial genomes from individual shotgun metagenomes
Source: Nucleic Acids Res. 2019 Mar 6;47(10):e57. doi: 10.1093/nar/gkz148 (PMC6547426; doi:10.1093/nar/gkz148)
Supplement: gkz148_Supplemental_Files [file gkz148_supplemental_files.zip › Autometa_SI.pdf]

Supplementary Data

Table S1. Genome sizes and DNA concentrations used to produce two synthetic metagenomes, “Mix-51-equal” and “Mix-51-staggered”, and to spike these into sponge metagenome “FL20-9”. See attached file: MIX-51_info.xlsx.

Table S2. Summary and accession information for all NCBI reference genomes used in the simulated Illumina sequencing for this study. See attached file**:** simulatedDatasets.xlsx

Table S3. Performance of automated binning programs compared to a previous semi-manual approach (Miller, Vanee, *et al.*, 2016) in recovery of the “*Candidatus* Endobugula sertula” from AB1_ovicells.

Method	Length (Mbp)	no. contigs	% "*Ca*. E. sertula" recovered (contigs, length)	completenes s	purity
Autometa	3.42	157	93.3% (92, 3,105,119 bp)	96.2	96.6
MaxBin	3.71	175	91.8% (88, 3,055,541 bp)	96.2	96.0
MetaBAT	2.9	90	86.6% (86, 2,881,097 bp)	93.2	98.0
MyCC	2.84	168	68.7% (73, 2,286,775 bp)	71.6	98.2
semi-manual	3.32	117	100% (117, 3,326,817 bp)	96.2	97.5

Table S4. Percentage of binned sequence by length based on kingdom level classification as inferred from Autometa’s Lowest Common Ancestor (LCA) workflow, in the AB1_ovicells dataset.

Method	Bacteria	Eukaryota	Viruses	Archaea	unclassified
Autometa w/ tax	100	0.0	0.0	0.0	0.0
Autometa w/out tax	97.7	0.8	0.1	0.0	1.4
MyCC	24.7	13.5	0.2	0.1	61.5
MaxBin	28.5	11.9	0.2	0.1	59.4
MetaBAT	24.6	13.2	0.2	0.1	61.9

Table S5. Results of MyCC binning for AB1_ovicells contigs that were unfiltered by Autometa's LCA workflow.

MyCC cluster	Size (Mbp)	N_{50}	No. contigs	CheckM completeness (%)	CheckM purity (%)	Coverage	GC (%)	CheckM lineage
Cluster.8	136.29	9923	16825	41.5	87.5	30.5	33.5	k__Archaea
Cluster.2	31.68	14997	2811	37.6	77.3	2.1	34.5	k__Archaea
Cluster.16	17.12	11618	1879	74.4	65.1	5.0	41.3	k__Archaea
Cluster.5	11.61	9846	1474	89.6	82.9	1.6	41.2	k__Bacteria
Cluster.1	7.76	33113	430	93.8	89.8	2.3	50.4	c__Gammaproteobacteria
Cluster.19	4.17	46272	231	92.6	92.7	2.0	60.4	f__Rhodobacteraceae
Cluster.14	3.45	20489	283	87.9	54.9	5.0	46.4	k__Bacteria
Cluster.11	3.42	6074	561	40.0	88.3	1.6	53.9	f__Rhodobacteraceae
Cluster.12	3.13	10960	354	24.1	94.8	4.2	43.8	k__Bacteria
Cluster.20	3.00	4960	609	34.0	98.8	1.3	50.8	k__Bacteria
Cluster.4	2.83	43116	168	71.6	98.2	4.4	40.2	c__Gammaproteobacteria
Cluster.18	2.65	6118	406	56.5	89.3	1.7	43.6	c__Gammaproteobacteria
Cluster.13	1.99	27349	126	98.7	100.0	26.6	34.3	k__Bacteria
Cluster.15	1.78	6310	303	67.9	97.7	1.5	40.1	c__Gammaproteobacteria
Cluster.22	1.31	4099	299	26.0	100.0	6.2	33.1	s__algicola
Cluster.10	1.06	4363	233	40.3	100.0	1.3	48.9	c__Gammaproteobacteria
Cluster.9	0.83	4168	195	16.7	100.0	1.2	49.3	root
Cluster.7	0.81	4491	172	17.8	99.6	1.4	49.3	c__Gammaproteobacteria
Cluster.6	0.72	4047	172	17.2	100.0	1.2	48.2	k__Bacteria
Cluster.3	0.70	593222	16	15.7	100.0	16.4	22.8	k__Bacteria
Cluster.21	0.65	17313	58	61.5	99.8	6.7	23.7	k__Bacteria
Cluster.17	0.60	16076	65	10.3	94.0	7.8	33.3	p__Cyanobacteria

Table S6. Length of sequence (in bp) based on kingdom level classification in MyCC bins that were unfiltered by Autometa's LCA workflow, in the AB1_ovicells dataset.

MyCC_cluster	Bacteria	Eukaryota	Viruses	Archaea	unclassified	total length
Cluster.8	3,129,421	23,629,421	38,514	299,748	109,189,222	136,286,326
Cluster.2	1,763,368	4,663,853	29,801	34,257	25,190,411	31,681,690
Cluster.16	2,480,635	3,342,491	61,185	40,085	11,198,545	17,122,941
Cluster.5	11,555,610	0	0	0	52,497	11,608,107
Cluster.1	7,702,631	5,550	0	0	51,469	7,759,650
Cluster.19	4,171,828	0	0	0	0	4,171,828
Cluster.14	3,413,645	0	0	11,925	28,344	3,453,914
Cluster.11	3,373,766	9,037	0	0	34,513	3,417,316
Cluster.12	3,067,560	3,490	0	28,361	33,458	3,132,869
Cluster.20	2,935,365	5,355	0	0	54,441	2,995,161
Cluster.4	2,786,399	8,449	0	3,288	34,683	2,832,819
Cluster.18	2,648,425	0	0	0	3,763	2,652,188
Cluster.13	1,777,789	140,579	0	0	72,184	1,990,552
Cluster.15	1,770,202	0	0	0	8,513	1,778,715
Cluster.22	1,254,005	15,479	0	27,945	11,970	1,309,399
Cluster.10	1,055,973	0	0	0	0	1,055,973
Cluster.9	829,401	0	0	0	0	829,401
Cluster.7	785,921	0	0	11,309	16,013	813,243
Cluster.6	718,005	0	0	0	0	718,005
Cluster.3	608,007	19,179	0	0	73,961	701,147
Cluster.21	538,243	35,539	0	0	78,722	652,504
Cluster.17	290,066	218,709	0	4,042	88,344	601,161

Table S7. Length-weighted coverages and percent genome coverages for the genomes of 51 human gut microbiome bacteria in Mix-51 datasets. See attached file: MIX-51_coverage_stats.xlsx.

Table S8. Assembly statistics for all 51 input genomes in the five Mix-51 assemblies. Contigs in assemblies were identified as belonging to one of the 51 reference genomes with MetaQUAST, and the identified contigs were assessed separately for each respective genome for assembly quality. See attached file: MIX51_assembly_stats.xlsx.

Table S9. F1 for each binning program in the Mix-51 datasets as determined by alignments to known reference genomes using metaQUAST (Mikheenko *et al.*, 2016). See attached file: MIX-51-F1-masters.xlsx.

Table S10. Median F1 and F1 recovery for bins obtained from the Mix-51 datasets. Note: the highest score in each row is underlined.

Dataset	Measure	Autometa	MyCC	MaxBin	MetaBAT	BusyBee
Mix-51	Median F1	<u>0.9163</u>	0.5609	0.7176	0.6791	0.3214
	F1 recovery	<u>0.809</u>	0.6196	0.6656	0.6616	0.4239
Mix-51-equal	Median F1	<u>0.9087</u>	0.8635	0.6787	0.7752	0.6291
	F1 recovery	<u>0.82</u>	0.7795	0.6258	0.7073	0.6677
Mix-51-staggered	Median F1	<u>0.8369</u>	0.7094	0.6981	0.7836	0.7094
	F1 recovery	0.6312	0.594	0.6024	<u>0.6568</u>	0.5847
FL20-9-Mix-51-equal	Median F1	<u>0.9011</u>	0.6272	0.6835	0.7421	*
	F1 recovery	<u>0.729</u>	0.6471	0.6277	0.6968	*
FL20-9-Mix-51-staggered	Median F1	<u>0.8522</u>	0.6775	0.6694	0.7232	*
	F1 recovery	<u>0.7082</u>	0.6078	0.5888	0.6612	*

*Input file sizes were too large for submission to BusyBee.

Table S11. Recall and precision values for *Bacteroides thetaiotaomicron* strains in Mix-51.

		Autometa	MyCC	MaxBin	MetaBAT	BusyBee
Bacteroides thetaiotaomicron 7330	**recall**	30	94	62	59	95
	**precision**	28	5	26	17	4
Bacteroides thetaiotaomicron VPI-5482	**recall**	29	95	70	64	97
	**precision**	41	8	45	28	6
Bacteroides thetaiotaomicron 3731	**recall**	24	93	37	38	97
	**precision**	29	6	20	14	5

Table S12. Total number of bins recovered by tested binning programs, where "NA" denotes cases where programs failed to complete successfully.

Method	78.125 Mbp	156.25 Mbp	312.5 Mbp	625 Mbp	1250 Mbp	2500 Mbp	5000 Mbp	10000 Mbp
Autometa	23	46	93	172	366	613	936	165
MyCC	23	42	81	139	275	315	NA	141
MaxBin	22	38	62	109	185	298	565	116
MetaBAT	21	42	75	118	233	347	NA	NA
BusyBee	20	30	61	NA	NA	NA	NA	NA

Table S13. Median F1 values for obtained bins, where "NA" denotes cases where programs failed to complete successfully.

Method	78.125 Mbp	156.25 Mbp	312.5 Mbp	625 Mbp	1250 Mbp	2500 Mbp	5000 Mbp	10000 Mbp
Autometa	1.00	0.98	0.99	0.98	0.97	0.95	0.88	0.02
MyCC	0.99	0.97	0.93	0.88	0.74	0.46	NA	0.02
MaxBin	0.98	0.94	0.91	0.67	0.42	0.17	0.23	0.02
MetaBAT	0.97	0.87	0.96	0.60	0.45	0.24	NA	NA

BusyBee	0.98	0.85	0.71	NA	NA	NA	NA	NA

Table S14. F1 recovery values for obtained bins, where “NA” denotes cases where programs failed to complete successfully.

Method	78.125 Mbp	156.25 Mbp	312.5 Mbp	625 Mbp	1250 Mbp	2500 Mbp	5000 Mbp	10000 Mbp
Autometa	0.95	0.91	0.92	0.89	0.87	0.77	0.61	0.04
MyCC	0.98	0.89	0.92	0.78	0.70	0.48	NA	0.06
MaxBin	0.86	0.79	0.66	0.55	0.46	0.37	0.33	0.04
MetaBAT	1.00	0.72	0.77	0.58	0.49	0.40	NA	NA
BusyBee	0.87	0.74	0.70	NA	NA	NA	NA	NA

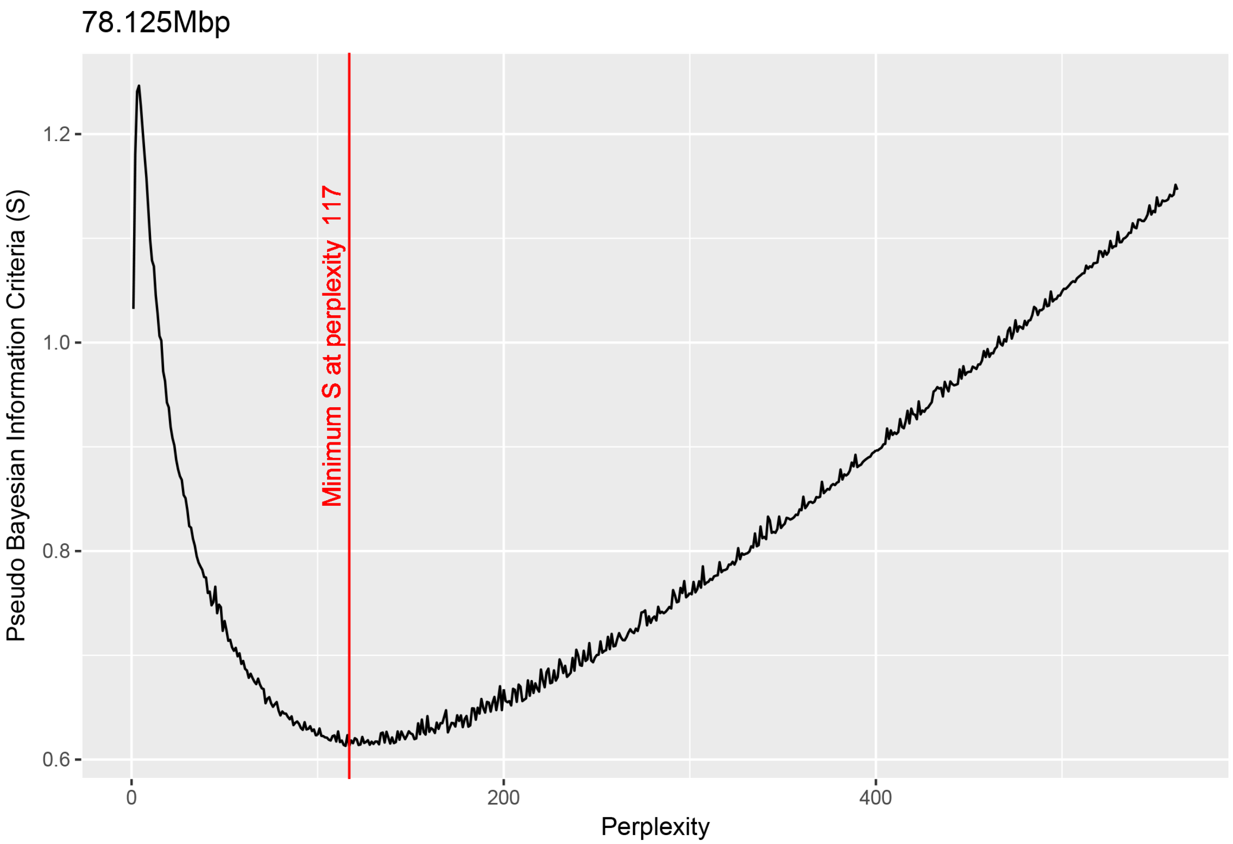

Figure S1. Plot of pseudo Bayesian Information Criteria (*S*) (Cao and Wang, 2017) against perplexity used in BH-tSNE for the simulated metagenome 78.125Mbp (see **Table 1**).

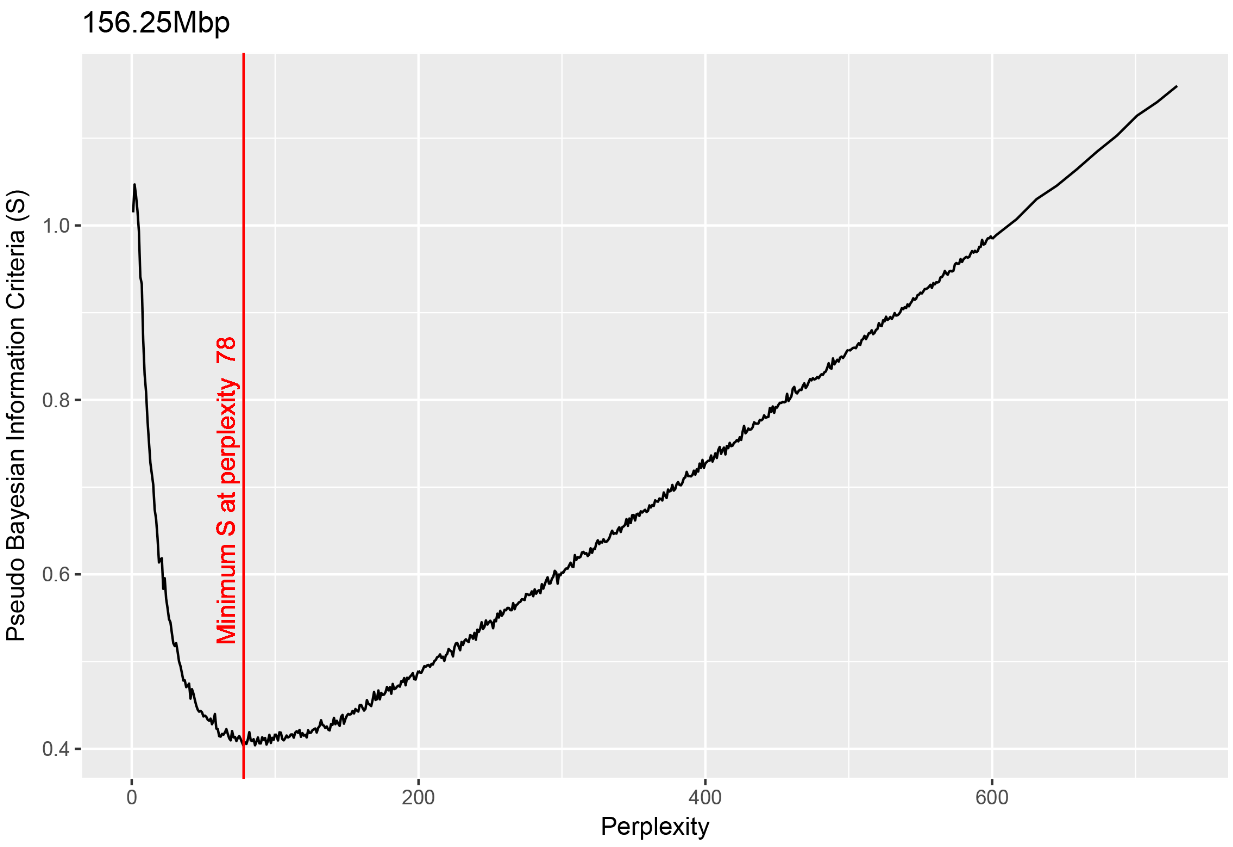

Figure S2. Plot of pseudo Bayesian Information Criteria (*S*) (Cao and Wang, 2017) against perplexity used in BH-tSNE for the simulated metagenome 156.25Mbp (see **Table 1**).

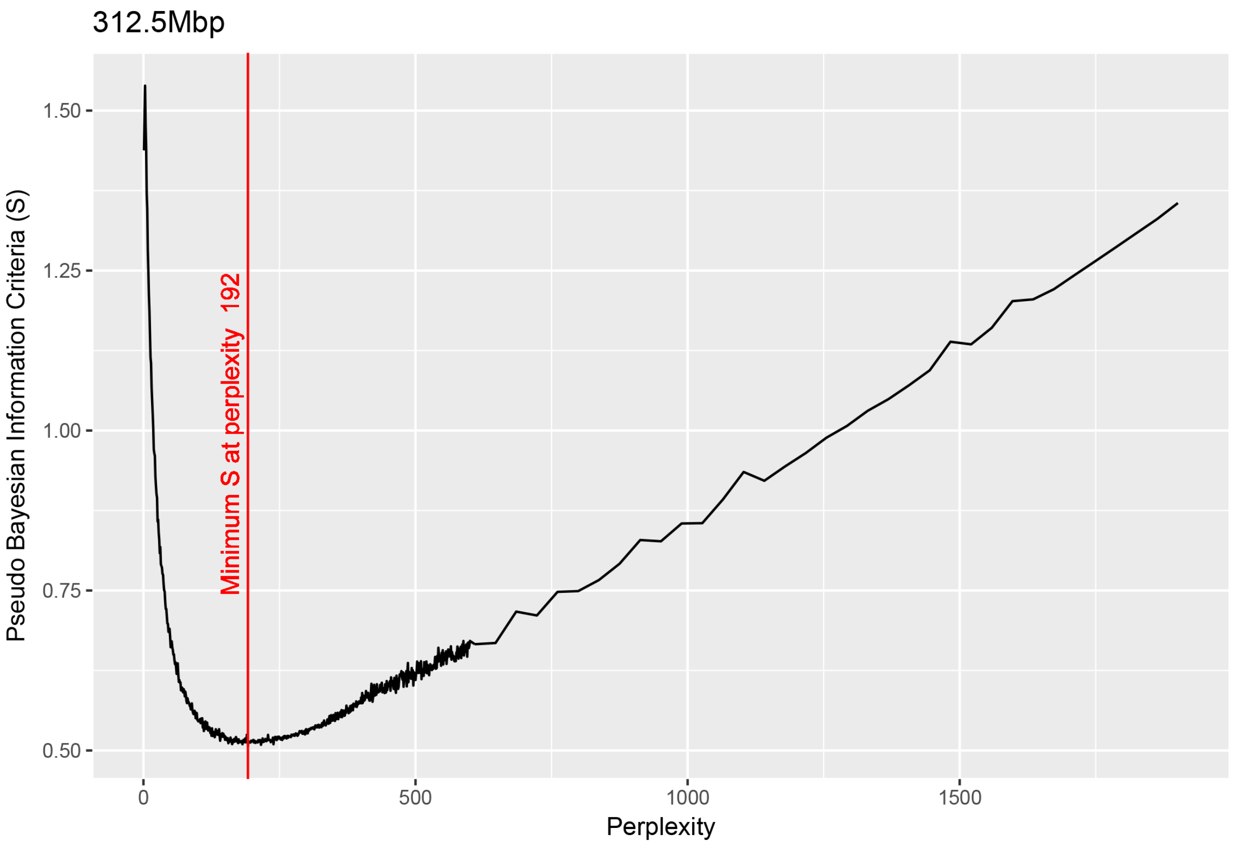

Figure S3. Plot of pseudo Bayesian Information Criteria (*S*) (Cao and Wang, 2017) against perplexity used in BH-tSNE for the simulated metagenome 312.5Mbp (see **Table 1**).

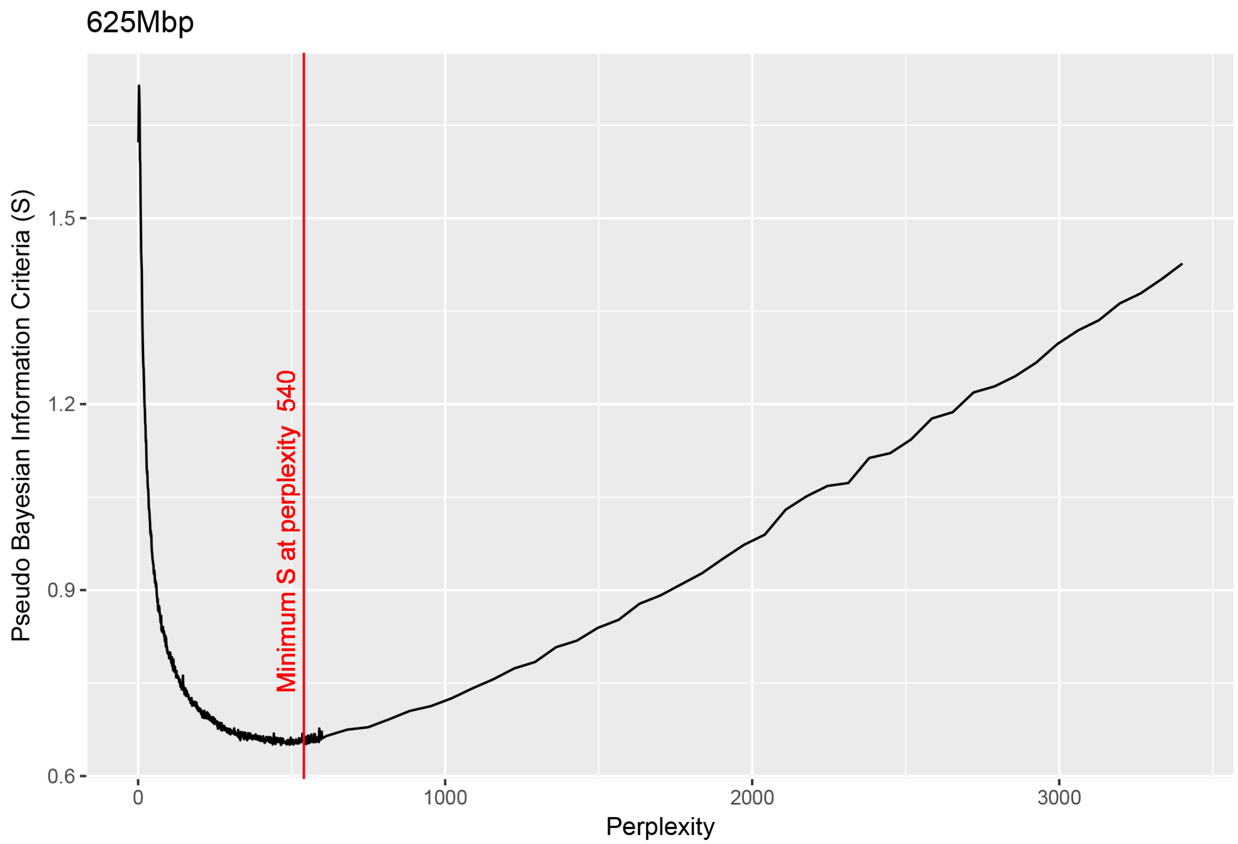

Figure S4. Plot of pseudo Bayesian Information Criteria (*S*) (Cao and Wang, 2017) against perplexity used in BH-tSNE for the simulated metagenome 625Mbp (see **Table 1**).

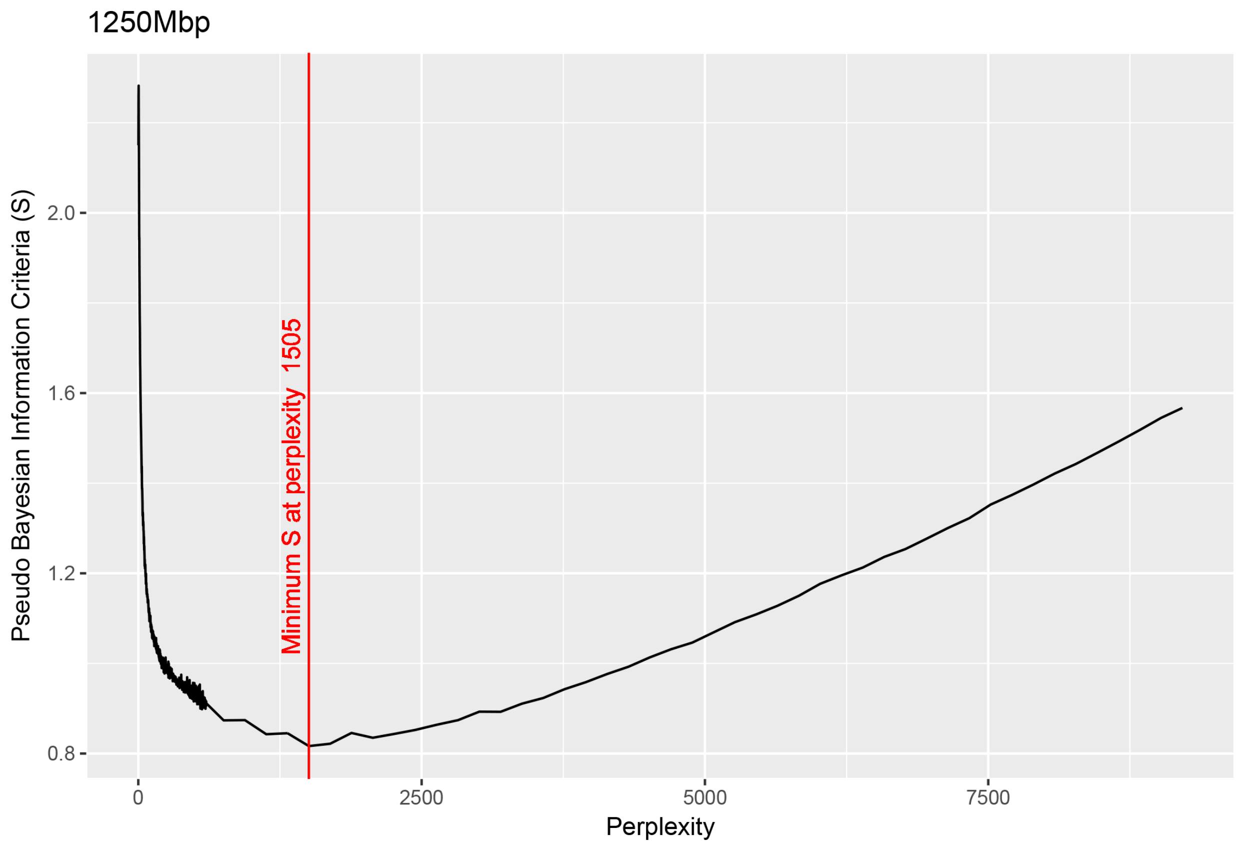

Figure S5. Plot of pseudo Bayesian Information Criteria (*S*) (Cao and Wang, 2017) against perplexity used in BH-tSNE for the simulated metagenome 1250Mbp (see **Table 1**).

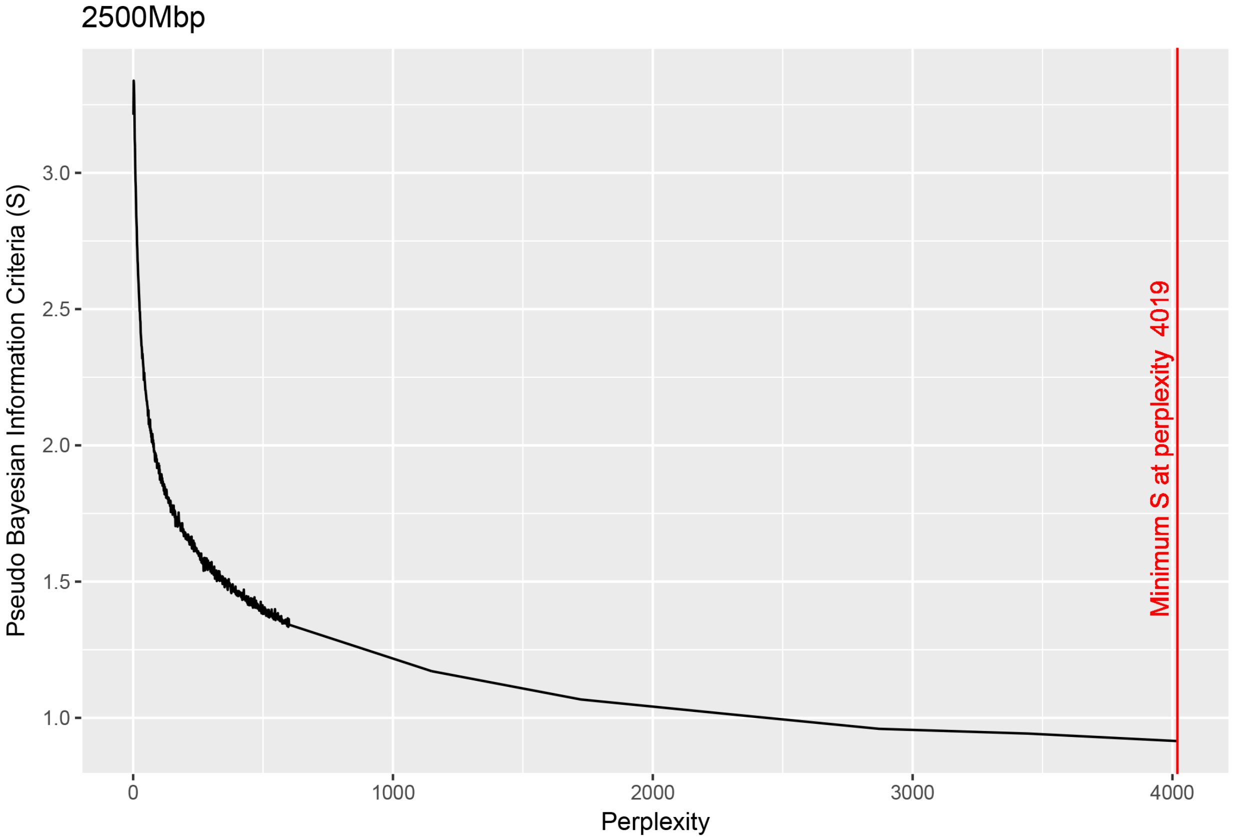

Figure S6. Plot of pseudo Bayesian Information Criteria (*S*) (Cao and Wang, 2017) against perplexity used in BH-tSNE for the simulated metagenome 2500Mbp (see **Table 1**).

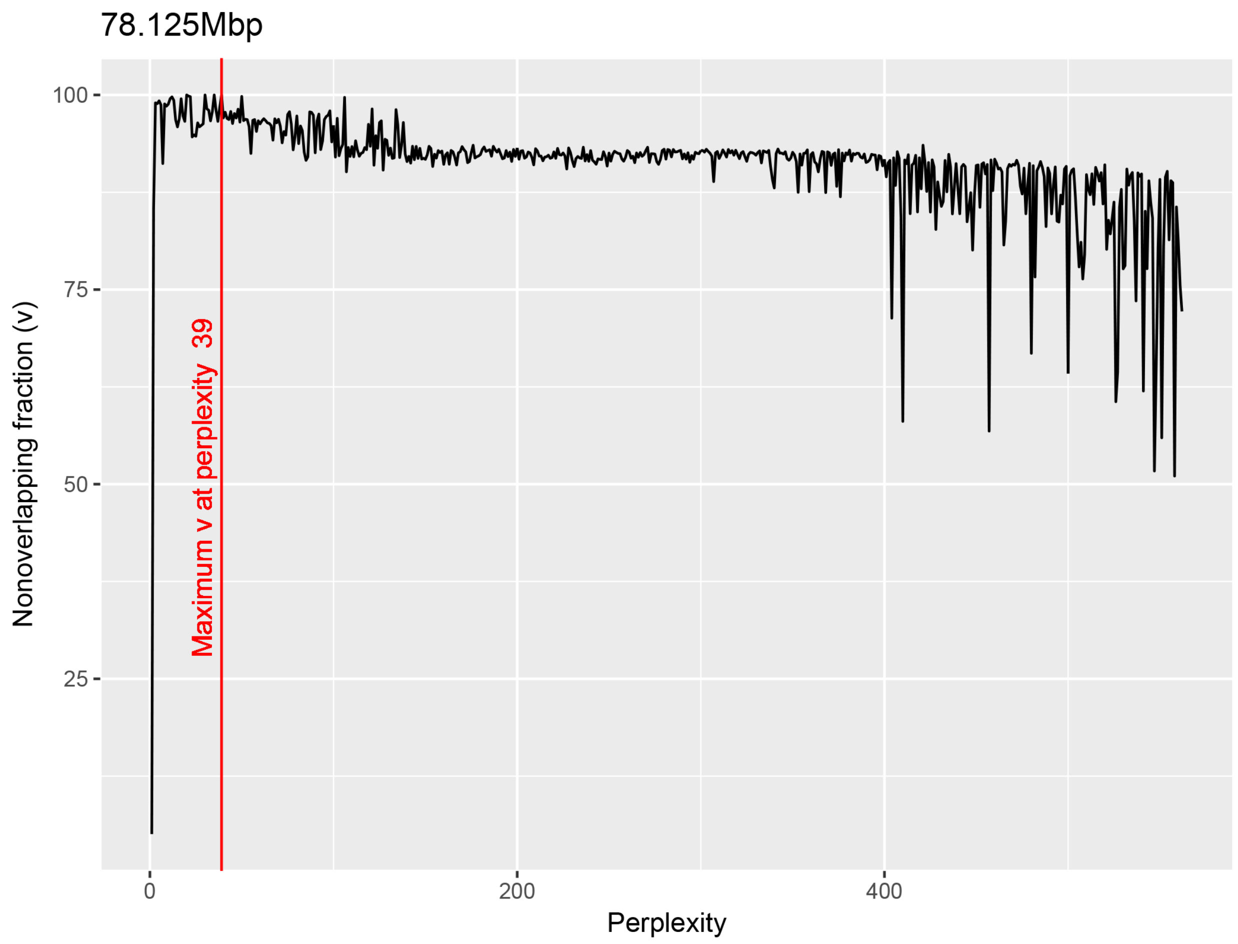

Figure S7. Plot of nonoverlapping fraction (*v*) against perplexity used in BH-tSNE for the simulated metagenome 78.125Mbp (see **Table 1**).

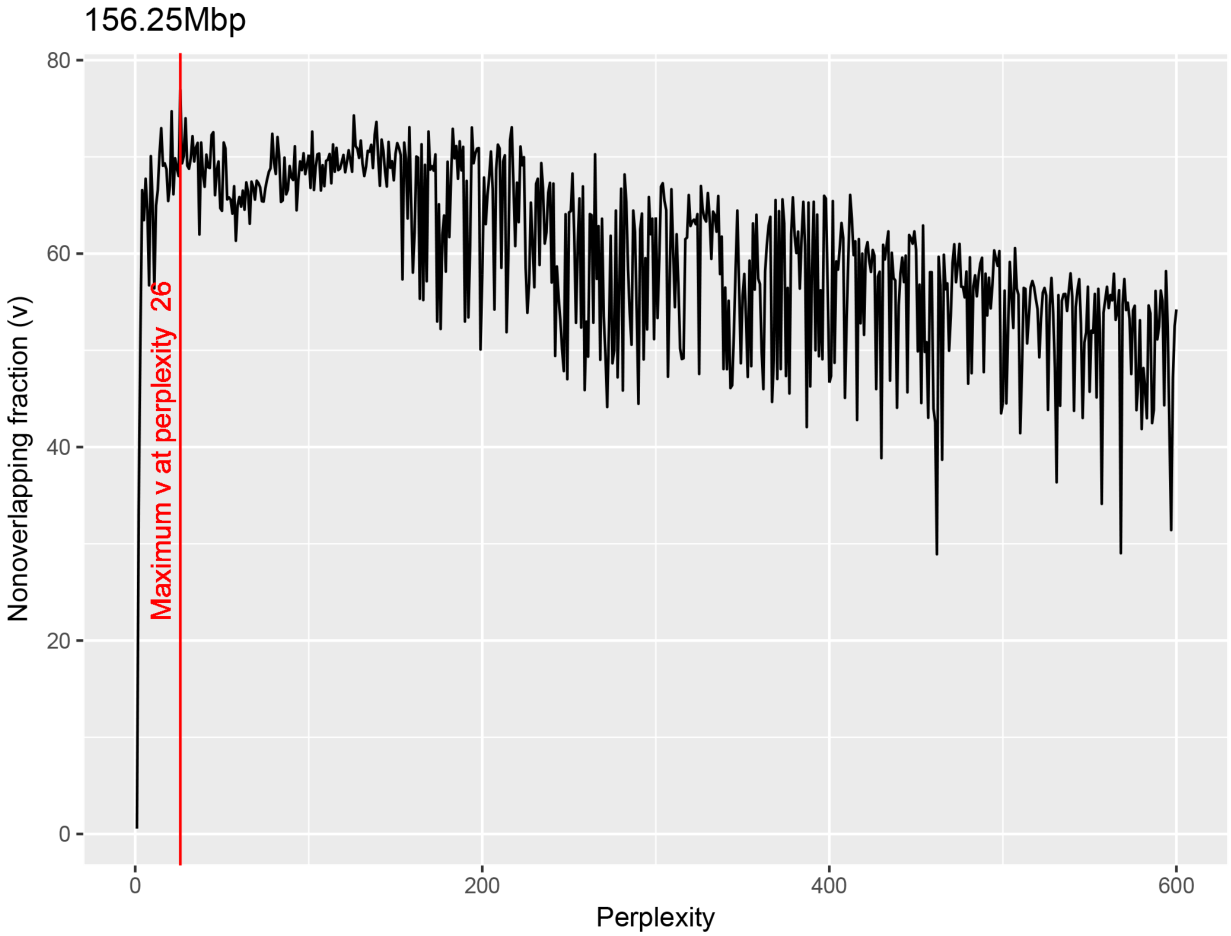

Figure S8. Plot of nonoverlapping fraction (*v*) against perplexity used in BH-tSNE for the simulated metagenome 156.25Mbp (see **Table 1**).

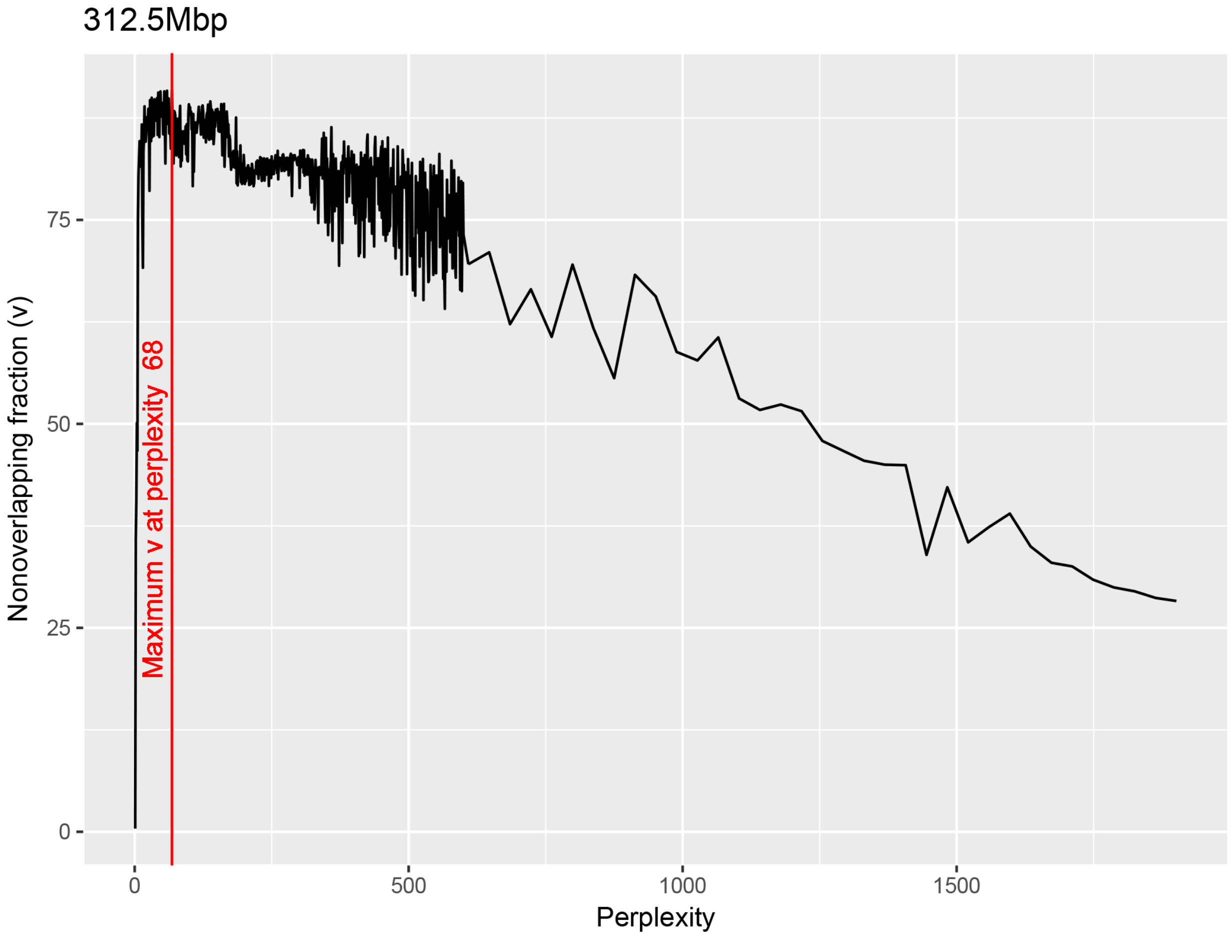

Figure S9. Plot of nonoverlapping fraction (*v*) against perplexity used in BH-tSNE for the simulated metagenome 312.5Mbp (see **Table 1**).

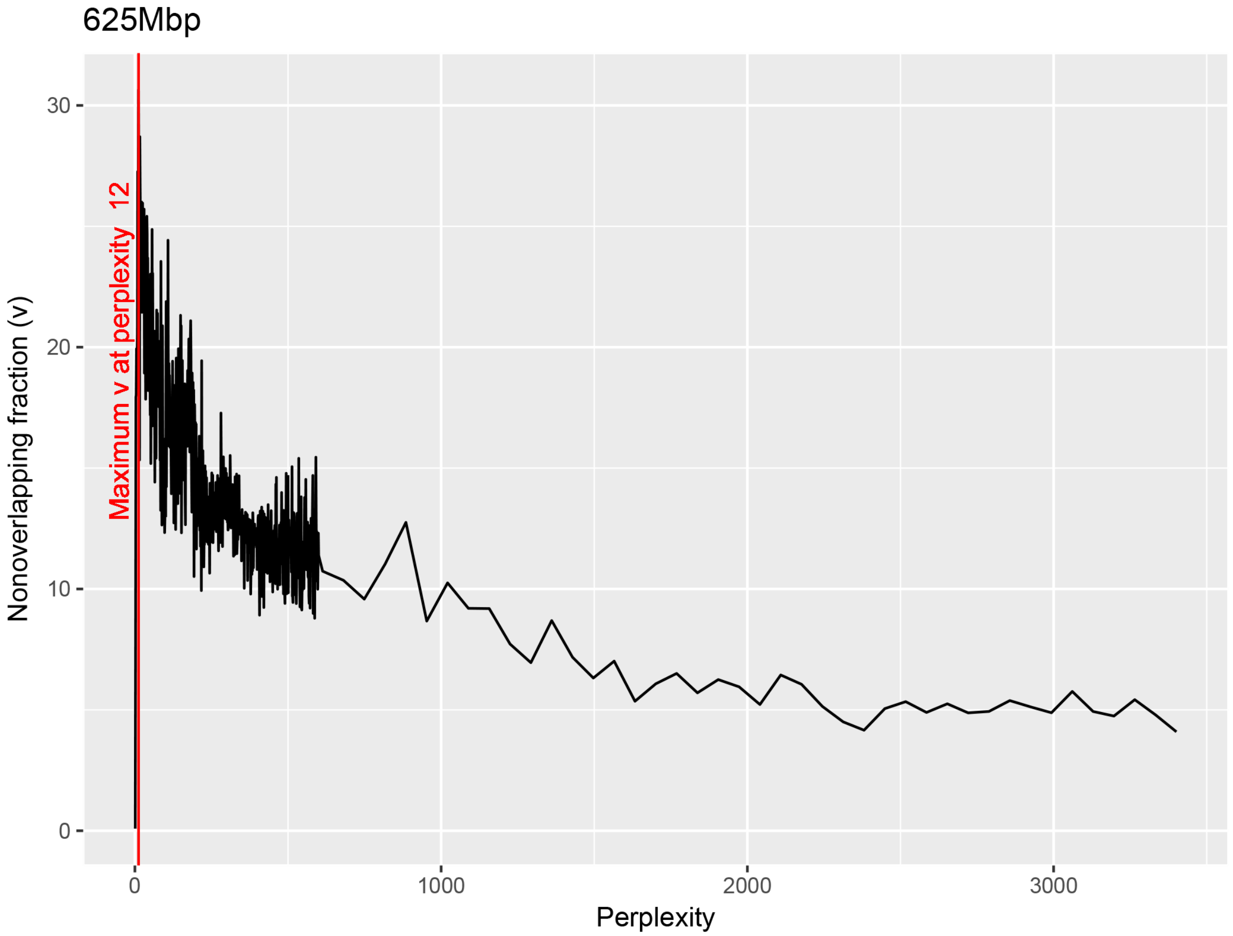

Figure S10. Plot of nonoverlapping fraction (*v*) against perplexity used in BH-tSNE for the simulated metagenome 625Mbp (see **Table 1**).

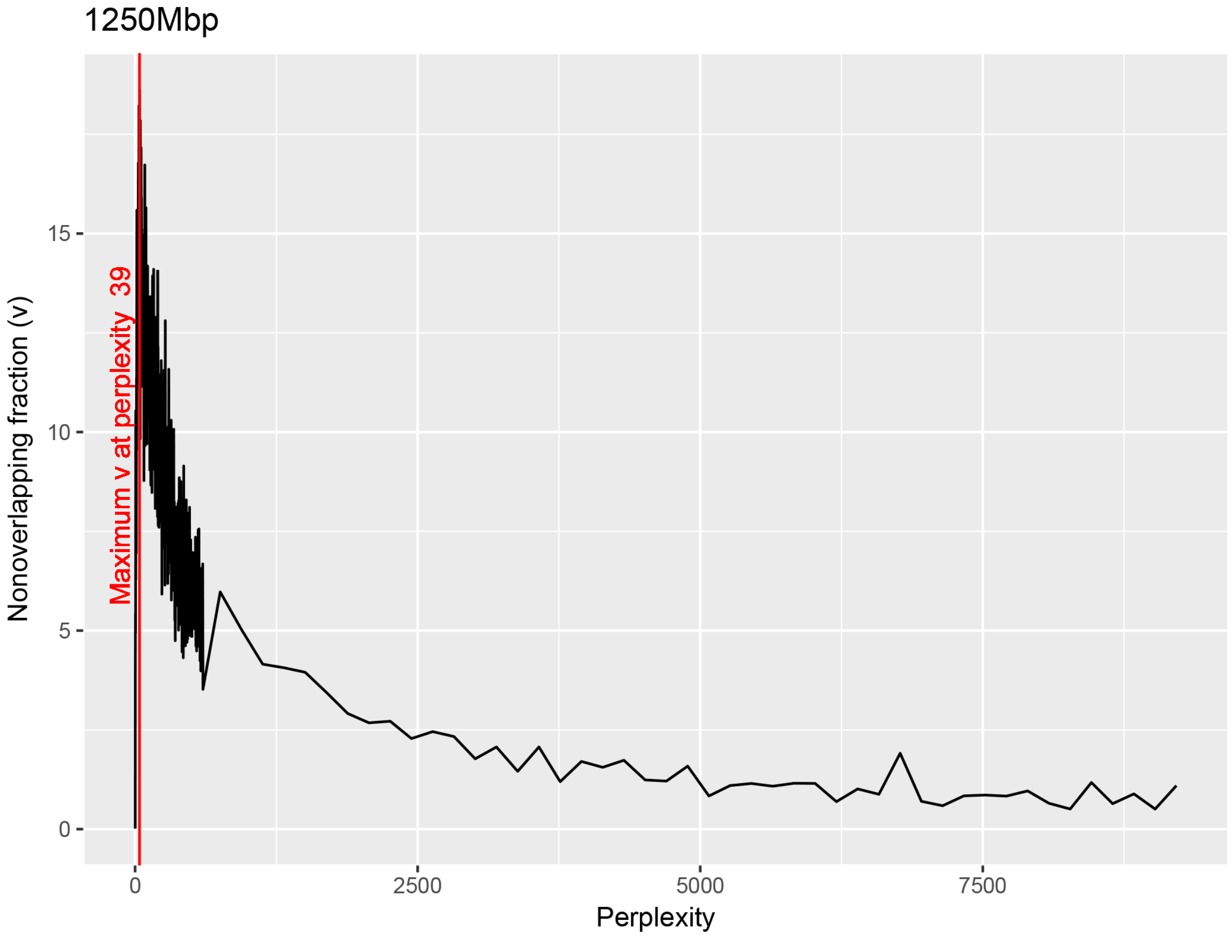

Figure S11. Plot of nonoverlapping fraction (*v*) against perplexity used in BH-tSNE for the simulated metagenome 1250Mbp (see **Table 1**).

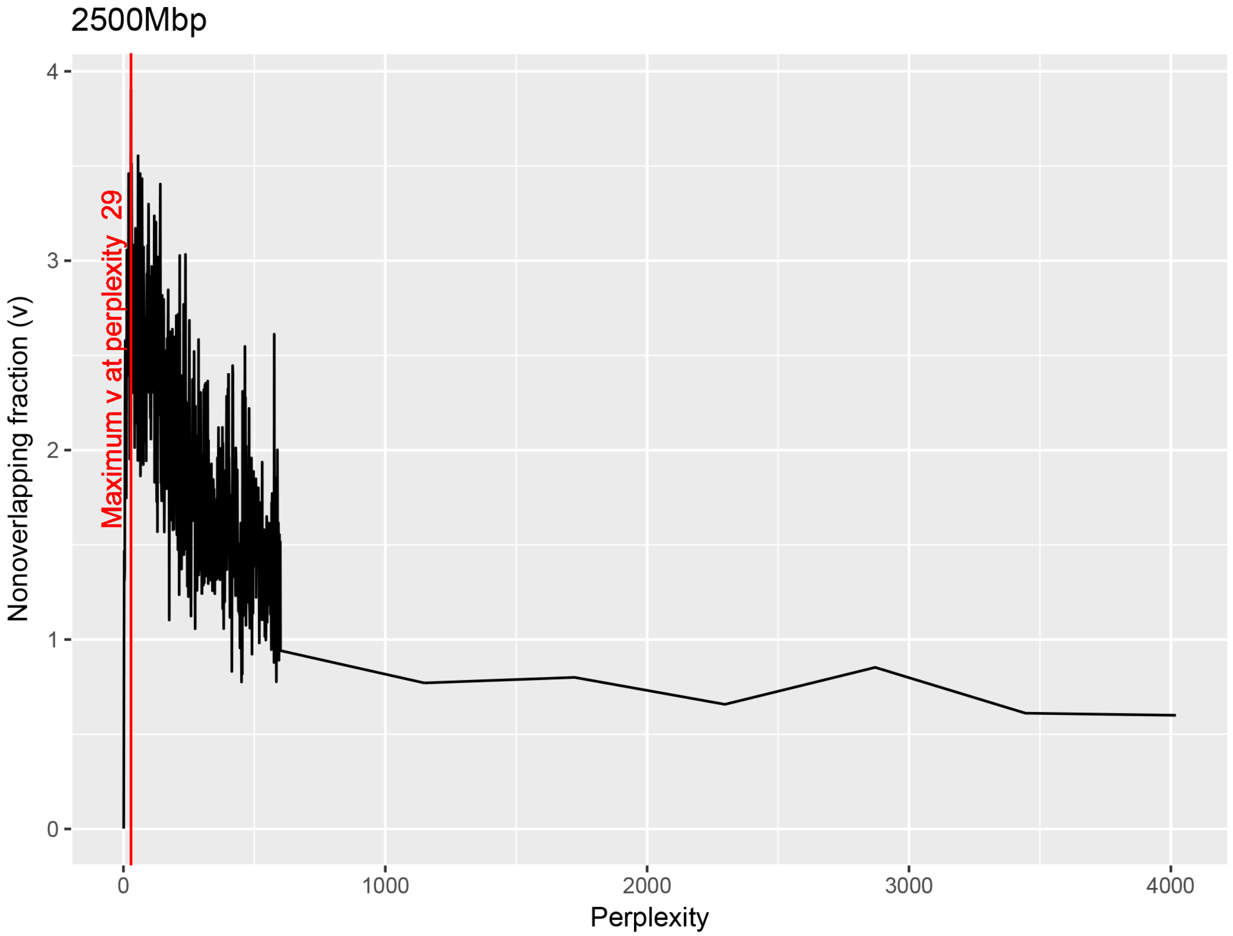

Figure S12. Plot of nonoverlapping fraction (*v*) against perplexity used in BH-tSNE for the simulated metagenome 2500Mbp (see **Table 1**).

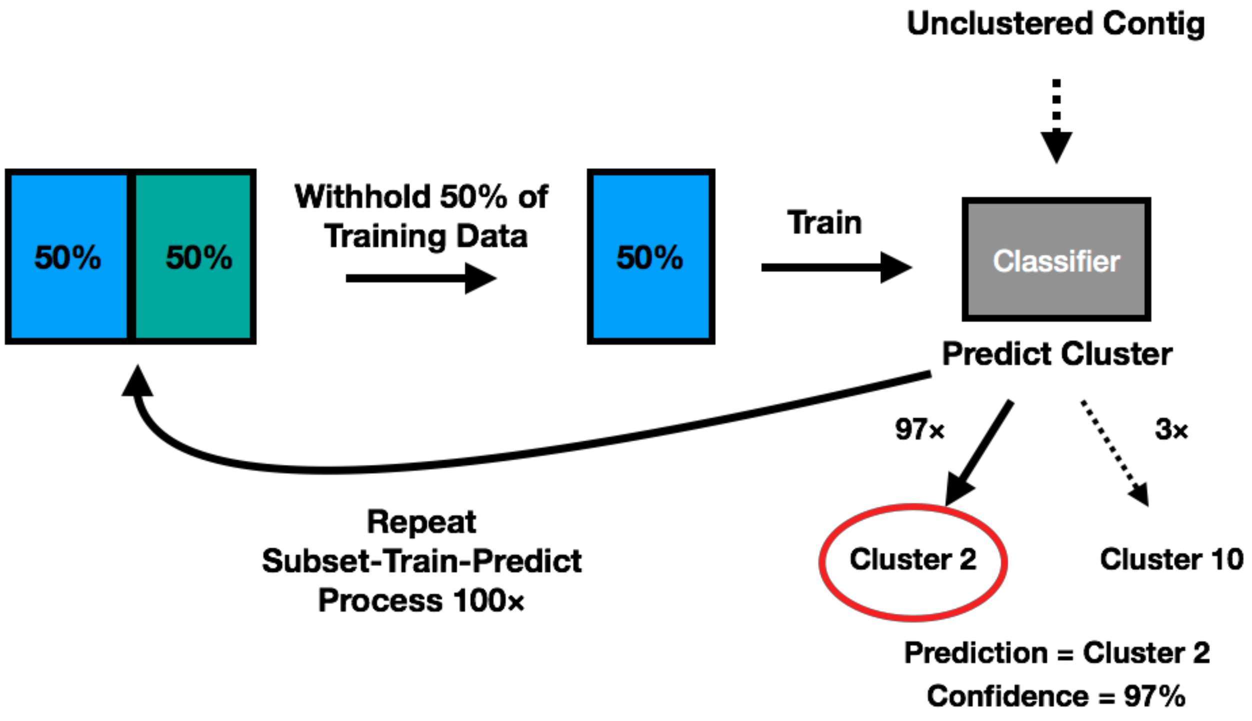

Figure S13. Schematic of jackknife cross validation concept, adapted from (Chevrette *et al.*, 2017).

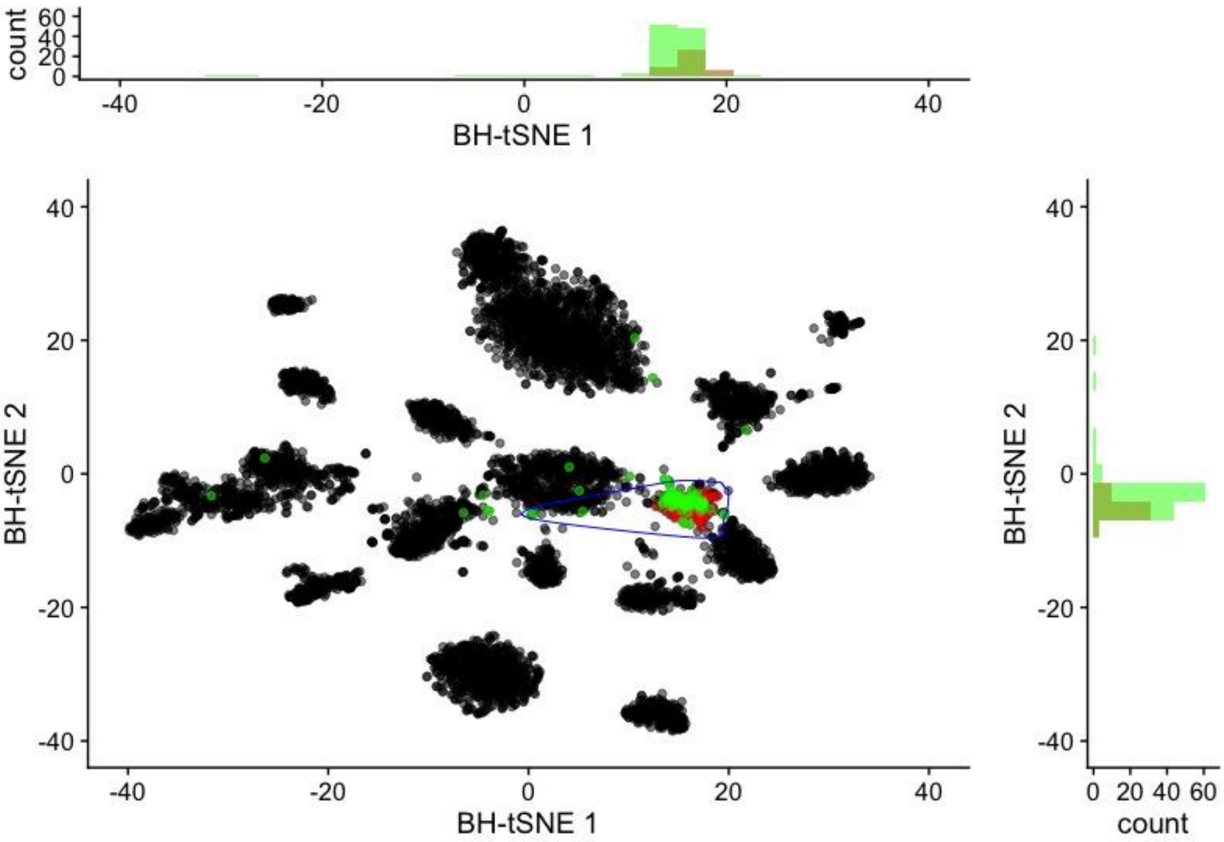

Figure S14. Nucleotide composition profiles of potential "*Ca.* E. sertula" contigs as identified by Autometa and MyCC. Contigs that were newly identified by both Autometa and MyCC as belonging to their "*Ca.* E. sertula" bin are colored in red whereas those that were previously classified as "*Ca.* E. sertula" by our previous semi-manual approach (Miller, Vanee, *et al.*, 2016; Miller, Weyna, *et al.*, 2016) are colored in green.

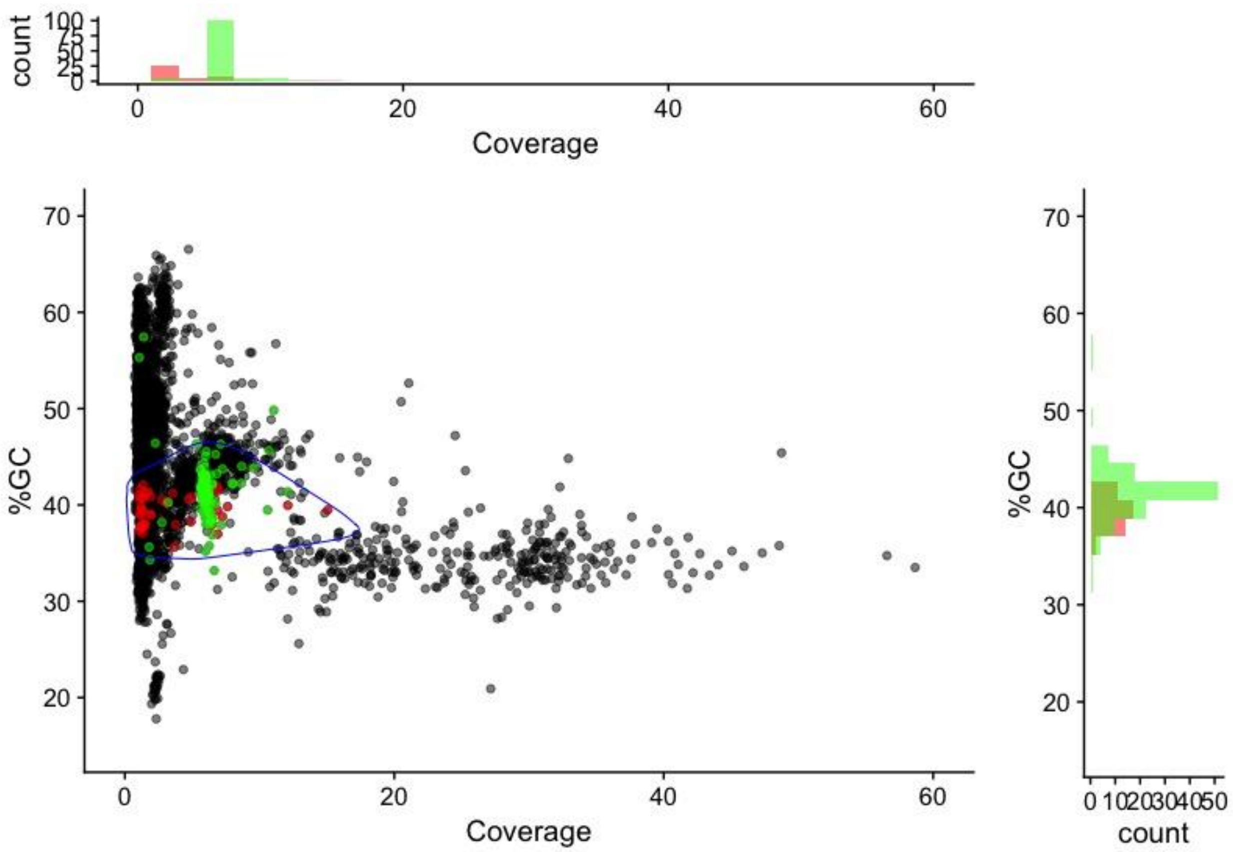

Figure S15. GC% and sequence coverage profiles of potential "*Ca.* E. sertula" contigs as identified by Autometa and MyCC. Contigs that were newly identified by both Autometa and MyCC as belonging to their "*Ca.* E. sertula" bin are colored in red whereas those that were previously classified as "*Ca.* E. sertula" by our previous semi-manual approach (Miller, Vanee, *et al.*, 2016; Miller, Weyna, *et al.*, 2016) are colored in green.

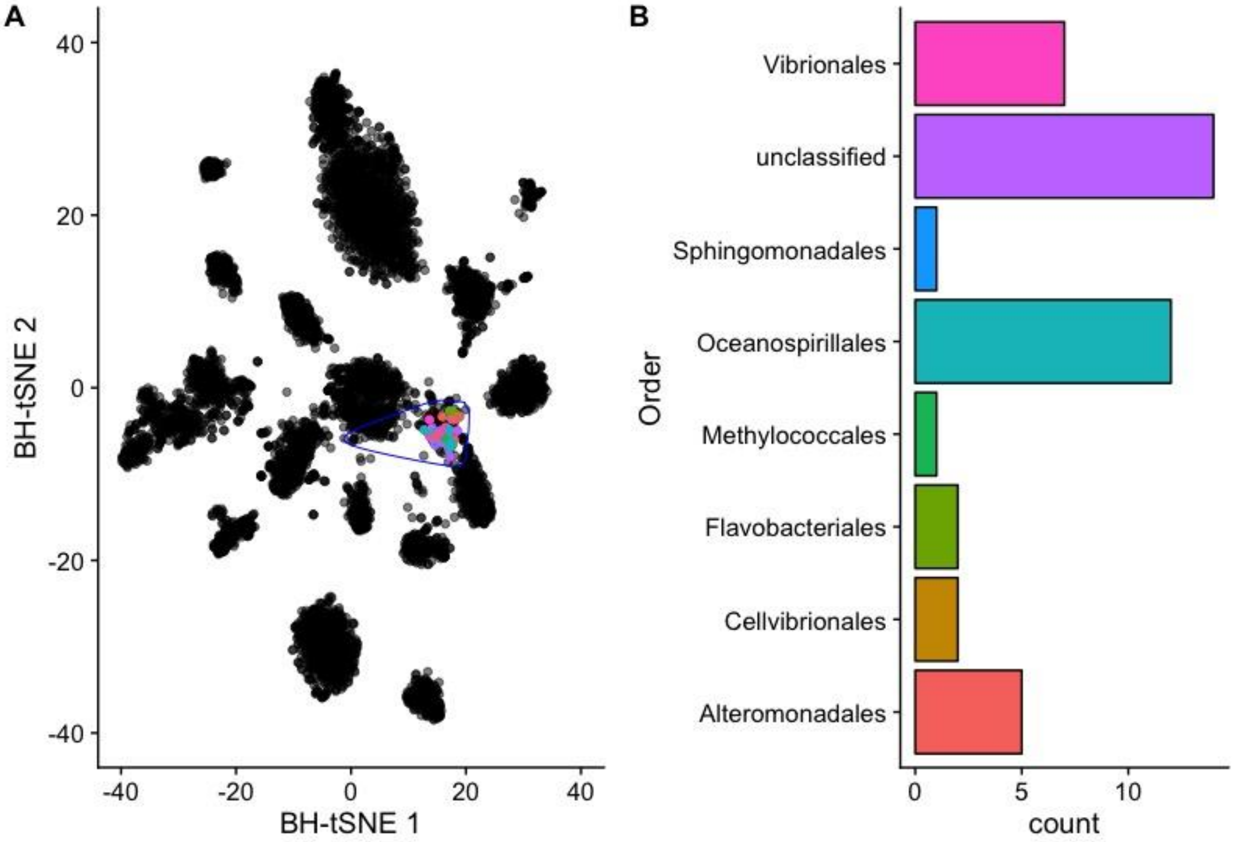

Figure S16. Taxonomic classification of potential "*Ca.* E. sertula" contigs as defined by Autometa's LCA workflow. Twelve of 44 contigs are classified as "Oceanospirillales" as the order level. Eleven of these 12 contigs are classified as "*Endozoicomonas*" at the genus level and thus likely represent contamination with the "AB1_endozoicomonas genome" bin we identified in a previous study by semi-manual binning approach (Miller, Weyna, *et al.*, 2016).

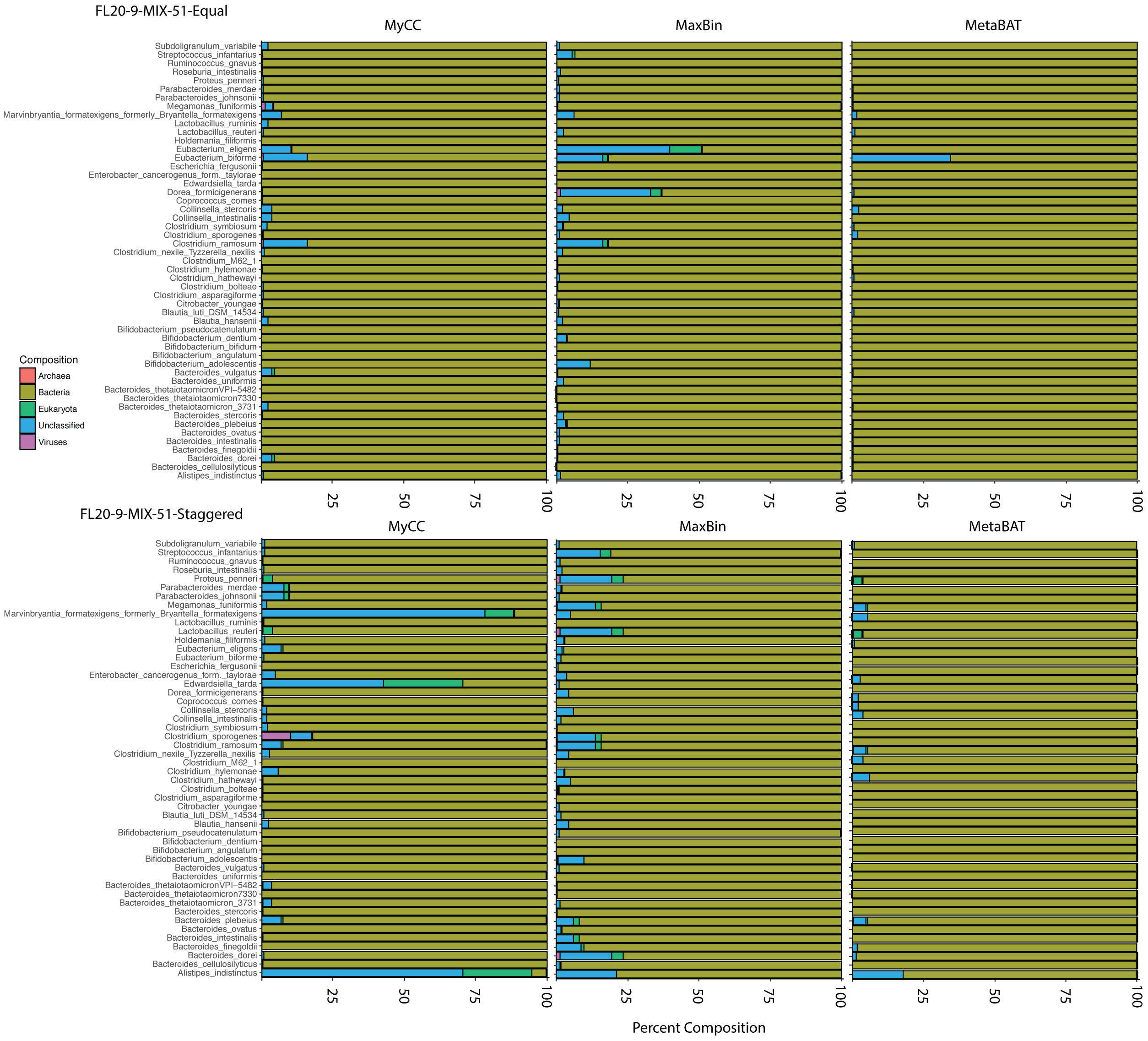

Figure S17. Eukaryotic contamination in the bins produced by MyCC, Maxbin and MetaBAT from the metagenomes FL20-9-Mix-51-equal and FL20-9-Mix-51-staggered.

Supplementary References

Cao,Y. and Wang,L. (2017) Automatic selection of t-SNE Perplexity. *arXiv:1708.03229 [cs.AI]*.

Chevrette,M.G. *et al.* (2017) SANDPUMA: Ensemble predictions of nonribosomal peptide chemistry reveals biosynthetic diversity across *Actinobacteria*. *Bioinformatics*, **33**, 3202–3210.

Mikheenko,A. *et al.* (2016) MetaQUAST: Evaluation of metagenome assemblies. *Bioinformatics*, **32**, 1088–1090.

Miller,I.J., Vanee,N., *et al.* (2016) Lack of overt genome reduction in the bryostatin-producing bryozoan symbiont '*Candidatus* Endobugula sertula'. *Appl. Environ. Microbiol.*, **82**, 6573–6583.

Miller,I.J., Weyna,T.R., *et al.* (2016) Single sample resolution of rare microbial dark matter in a marine invertebrate metagenome. *Sci. Rep.*, **6**, 34362.

Nurk,S. *et al.* (2017) metaSPAdes: A new versatile metagenomic assembler. *Genome Res.*, **27**, 824–834.
